# Prediction of Mortality by Clinical Laboratory Parameters in Severe Fever with Thrombocytopenia Syndrome: A Meta-Analysis

**DOI:** 10.3390/tropicalmed10070193

**Published:** 2025-07-09

**Authors:** Shicui Yan, Xuebin Ding, Qiao Gao, Lili Zhao, Cong Li, Zhenlu Sun, Xuejun Ma

**Affiliations:** 1 Yantai Center for Disease Control and Prevention, No. 17 Fuhou Road, Laishan District, Yantai 264003, China; 2 National Key Laboratory of Intelligent Tracking and Forecasting for Infectious Diseases, NHC Key Laboratory of Medical Virology and Viral Diseases, National Institute for Viral Disease Control and Prevention, Chinese Center for Disease Control and Prevention, No. 155, Changbai Street, Changping District, Beijing 102206, China

**Keywords:** SFTS, laboratory parameters, mortality, meta-analysis

## Abstract

Background: This study intended to fully assess the predictive efficiency of different clinical laboratory parameters for the mortality risk in severe fever with thrombocytopenia syndrome (SFTS). Methods: We systematically searched the Web of Science, PubMed, Cochrane Library, and Embase up to 13 December 2024 for studies on the association of laboratory parameters with SFTS mortality. Two investigators were independently responsible for the study screening and data extraction, and they assessed the study quality using the Newcastle–Ottawa Scale (NOS). Stata17.0 was adopted for the meta-analyses. Results: We finally included 33 observational studies involving 9502 participants (1799 deaths and 7703 survivors). The results showed that increases in the viral load (odds ratio (OR) 1.93, 95% confidence interval (CI) 1.56–2.38), neutrophil-to-lymphocyte ratio (hazard ratio (HR) 1.31, 95% CI 1.13–1.51), neutrophil percentage (HR 1.02, 95% CI 1.01–1.03), white blood cells (HR 1.06, 95% CI 1.01–1.11), activated partial thromboplastin time (OR 1.07, 95% CI 1.04–1.09), prothrombin time (OR 1.31, 95% CI 1.03–1.65), creatine kinase-myocardial band (OR 1.01, 95% CI 1.01–1.02), and procalcitonin (HR 1.27, 95% CI 1.10–1.47) greatly increased the SFTS mortality, while decreases in the lymphocyte percentage (HR 0.96, 95% CI 0.94–0.98), platelets (HR 0.98, 95% CI 0.97–0.99), and albumin (HR 0.91, 95% CI 0.86–0.96) also greatly increased the SFTS mortality; the results were all statistically significant (*p* < 0.05). Conclusion: Abnormalities of laboratory parameters (e.g., viral load, blood routine, coagulation, multi-organ dysfunction, and inflammation indicators) are good predictors of SFTS mortality, which can provide valuable references in clinical practice.

## 1. Introduction

Severe fever with thrombocytopenia syndrome (SFTS), a novel tick-borne acute infectious disease that may be caused by SFTS virus (SFTSV), presents with high fever, debilitation, severe thrombocytopenia, gastrointestinal and central nervous system symptoms, and even multi-organ dysfunction. SFTS can rapidly develop into multi-organ failure in some cases, with a fatality rate of 7.80% (95% CI 7.01–8.69%) and a mortality rate of 3.49 (95% CI 2.97–4.10) per ten million people [1], imposing a heavy social burden. Since 2009, SFTS has been identified in over 20 provinces in China, and cases of infections have also been reported in Republic of Korea [2], Japan [3,4], Vietnam [5], Pakistan [6], Myanmar [7], and Thailand [8,9]. Due to the annually rising morbidity and mortality rates worldwide, SFTS has presented a serious challenge to public health. In 2018, SFTS was identified by the World Health Organization as a priority disease for medical research in emergency contexts [10]. However, effective antiviral drugs or vaccines specifically for SFTS prevention and treatment are lacking nowadays [11], and SFTS has become one of the major infectious diseases threatening public health. Therefore, identifying reliable biomarkers for the early prediction of SFTS patients at high risk of mortality is crucial for clinical management and can help in the design of personalized treatments to raise the survival rate.

Multiple previous meta-analyses have systematically investigated the risk factors for SFTS mortality, such as advanced age, central nervous system symptoms, bleeding tendency, high viral loads, and elevated serum enzymes [12]. Moreover, death cases are more prone to multi-organ failure, disseminated intravascular coagulation, and severe arrhythmia [13]. The mortality risk also significantly increases due to hospitalization delays, underlying diseases, and comorbidities [14]. However, these findings are mostly based on subjective clinical assessments or composite outcome measures. Notably, research suggests that routine laboratory parameters can act as objective prognostic markers. Chen [15] argued that great decreases in platelet (PLT) and albumin (ALB) and increases in aspartate aminotransferase (AST), activated partial thromboplastin time (APTT), and creatine kinase (CK) levels are strongly correlated with the mortality risk. Wang [16], by analyzing the standardized mean difference (SMD), further verified the notable differences in viral load, coagulation function, liver and kidney function, and myocardial injury indicators between the survivors and deaths. However, the prognostic value of laboratory parameters in SFTS remains controversial, which is attributed primarily to the differences in inclusion criteria, heterogeneity in study design, limitation of the sample sizes, and inconsistency of the statistical methods. Therefore, large-sample multicenter studies and standardized analytical frameworks are required for further validation.

Clinical laboratory parameters possess significant advantages, since they are objective and quantifiable and can be dynamically monitored. Therefore, we synthesized the available data on the association of laboratory parameters with SFTS mortality to evaluate the predictive efficiency of different parameters for the mortality risk. Compared with previous studies [17], this study included larger sample sizes and more laboratory parameters, with the hazard ratio (HR) or odds ratio (OR) as the effect size to more accurately quantify the predictive efficiency of laboratory parameters. We augmented the evidence base on laboratory predictors of SFTS mortality. Our analysis validated that abnormalities in viral load, blood routine, coagulation, liver function, and myocardial function indicators, along with systemic inflammation markers, demonstrated significant predictive utility for fatal outcomes. The findings will help prioritize the enhanced clinical monitoring of high-risk groups or in exploring personalized supportive strategies.

## 2. Materials and Methods

### 2.1. Protocol and Registration

This study adhered to the PRISMA statement (Table S1) [18], and the protocol was registered with PROSPERO in February 2025 (CRD42025644233).

### 2.2. Study Search

We systematically searched the Web of Science, PubMed, Cochrane Library, and Embase up to 13 December 2024 for studies on the association of laboratory parameters with SFTS mortality. Medical subject headings plus free terms were used, restricted to English terms including “severe fever with thrombocytopenia syndrome”, “Dabie bandavirus”, and “bunyavirus” (disease-related terms); and white blood cell (WBC), PLT, AST, alanine aminotransferase (ALT), ferritin, D-dimer (DD), and C-reactive protein (CRP) (laboratory parameter-related terms) (Table S2).

### 2.3. Eligibility Criteria

We established the eligibility criteria based on the PICOS principle. Inclusion criteria: (i) participant: patients with SFTS; (ii) laboratory parameters and mortality risk were reported, with HR or OR and 95% confidence interval (CI) calculated; laboratory parameters were initial data collected first at the time of admission; (iii) observational studies (cohort and case control studies); (iv) English studies.

Exclusion criteria: duplicate publications, meta-analyses, reviews, conference abstracts, case reports, reply letters, animal experiments, and studies with inappropriate design and objectives and no relevant data.

### 2.4. Study Screening and Data Extraction

The study screening, data extraction, and cross-checking were independently conducted by two investigators. Any discrepancy was resolved via discussion or consultation with a third investigator. After the retrieved studies were imported into EndNoteX9, the title and abstract were first read to exclude irrelevant studies. Then, the full text of the remainder was reviewed to finally include the eligible studies. The following data were extracted: first author, year of publication, study type, study design, study site, number of cases (survivors and deaths), and clinical laboratory parameters. The HR or OR with 95% CI for the association of parameters with SFTS mortality was extracted directly from multivariate or univariate regression analyses, of which the former was preferred if relevant data were provided by both analyses.

### 2.5. Quality Assessment

Two investigators assessed the study quality using the Newcastle–Ottawa Scale (NOS) [19] from eight questions in three domains (selection, comparability, and exposure) for case control studies and in three domains (selection, comparability, and outcome) for cohort studies. Each study was rated as high (≥7), medium (4–6), or low quality (1–4).

### 2.6. Data Analysis

Stata17.0 was utilized for the meta-analyses. We assessed the heterogeneity of the included studies using the I^2^ statistic. A random-effects model was employed when great heterogeneity was present (I^2^ > 50%); otherwise, a fixed-effects model was employed [20]. The effect size HR or OR with 95% CI for laboratory parameters was pooled, based on which the association of clinical laboratory parameters with SFTS mortality was analyzed and the predictive efficiency of the parameters was assessed. Subgroup analyses were performed by study type (retrospective or prospective), country (China orRepublic of Korea), and study site (one, two, or muti) to identify the source of heterogeneity. Leave-one-out sensitivity analyses were conducted for the robustness of the findings. Publication bias was assessed using an Egger’s test and funnel plot when at least five studies were included, and *p* < 0.05 was deemed significant publication bias. The bias was further corrected using the trim-and-fill method.

## 3. Results

### 3.1. Search Results

**Figure 1:**
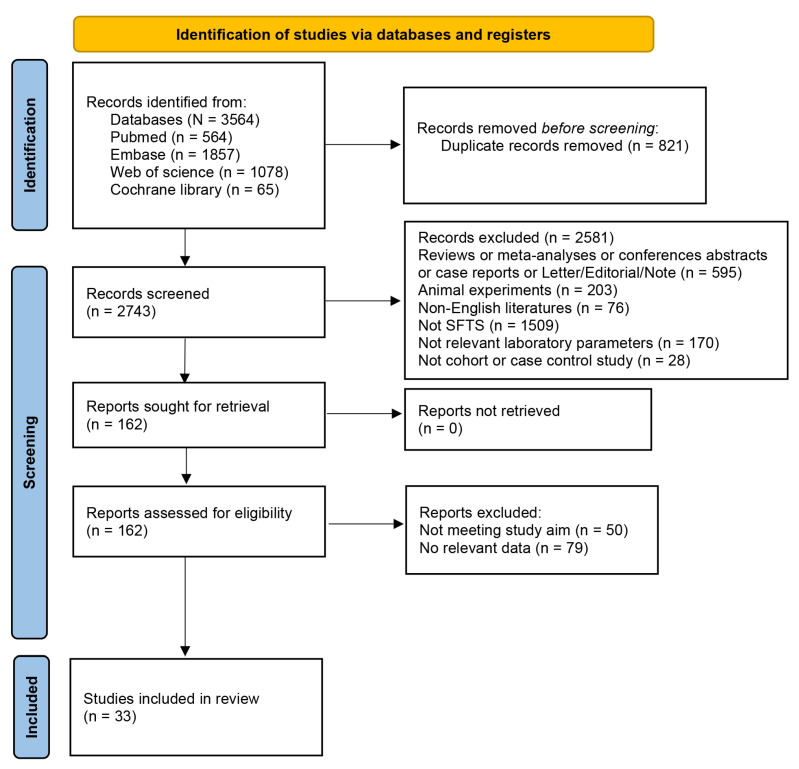
PRISMA 2020 flow diagram.

We initially retrieved 3564 relevant studies, of which 821 duplicates were excluded. After reviewing the title and abstract, 2581 studies were excluded due to inappropriate study design, participants, and exposure. Then, the full text of the remainder was examined, and we further excluded 129 studies due to inappropriate study objectives and having no relevant data. Finally, 33 studies were included from the total of 3564 studies (Figure 1).

### 3.2. Study Characteristics and Quality

The included studies [21,22,23,24,25,26,27,28,29,30,31,32,33,34,35,36,37,38,39,40,41,42,43,44,45,46,47,48,49,50,51,52,53] involved 9502 SFTS patients (7703 survivors and 1799 deaths), of which 6.06% (2/33) were prospective studies and 93.94% (31/33) were retrospective studies. They were all conducted in the Western Pacific region, specifically in China (32/33, 96.97%) and Republic of Korea (1/33, 3.03%) (Table 1).

The NOS score was >7 for all the included studies, suggesting high quality (Tables S3 and S4).

### 3.3. Meta-Analyses

#### 3.3.1. Association of Viral Load with SFTS Mortality

Great heterogeneity was found in the viral load results (I^2^ = 61.1%, *p* = 0.017), so a random-effects model was employed. The results showed that elevation of the viral load could effectively predict SFTS mortality (OR 1.93, 95% CI 1.56–2.38), displaying statistical significance (*p* < 0.05) (Figure S1).

#### 3.3.2. Association of Blood Routine Indicators with SFTS Mortality

No heterogeneity was found in the lymphocyte percentage (LYM%) (I^2^ = 0.0%, *p* = 1.000), neutrophil percentage (NEU%) (I^2^ = 0.0%, *p* = 0.834), or red blood cell (RBC) (I^2^ = 0.0%, *p* = 0.489) results, so a fixed-effects model was employed; great heterogeneity was found in the neutrophil-to-lymphocyte ratio (NLR) (I^2^ = 79.3%, *p* = 0.008), lymphocyte (LYM) (I^2^ = 65.0%, *p* = 0.014), neutrophil (NEU) (I^2^ = 83.0%, *p* = 0.000), monocyte (MONO) (I^2^ = 62.4%, *p* = 0.047), PLT (I^2^ = 70.4%, *p* = 0.000), hemoglobin (Hb) (I^2^ = 70.7%, *p* = 0.017), and WBC (I^2^ = 69.3%, *p* = 0.003) results, so a random-effects model was employed. The results showed that elevation of the NLR (HR 1.31, 95% CI 1.13–1.51), NEU% (HR 1.02, 95% CI 1.01–1.03), and WBC (HR 1.06, 95% CI 1.01–1.11) levels and decreases in LYM% (HR 0.96, 95% CI 0.94–0.98) and PLT (HR 0.98, 95% CI 0.97–0.99) could effectively predict SFTS mortality, displaying statistical significance (*p* < 0.05). The other blood routine indicators showed no statistical significance; thus, they could not yet be considered effective predictors for SFTS mortality (Figure S2).

#### 3.3.3. Association of Coagulation Indicators with SFTS Mortality

A random-effects model was adopted for the APTT (I^2^ = 68.1%, *p* = 0.003), thrombin time (TT) (I^2^ = 95.4%, *p* = 0.000), prothrombin time (PT) (I^2^ = 88.1%, *p* = 0.000), and fibrinogen (FIB) (I^2^ = 92.0%, *p* = 0.000) results due to their great heterogeneity. The results showed that elevation of the APTT (OR 1.07, 95% CI 1.04–1.09), TT (OR 1.24, 95% CI 1.06–1.44), and PT (OR 1.31, 95% CI 1.03–1.65) levels was a good predictor for SFTS mortality, showing statistical significance (*p* < 0.05). In contrast, FIB showed no statistical significance; thus, it could not yet be considered an effective predictor for SFTS mortality (Figure S3).

#### 3.3.4. Association of Liver Function Indicators with SFTS Mortality

A fixed-effects model was adopted for gamma-glutamyl transferase (GGT) (I^2^ = 17.5%, *p* = 0.303) due to its low heterogeneity, and a random-effects model for the alkaline phosphatase (ALP) (I^2^ = 62.4%, *p* = 0.031), AST (I^2^ = 98.1%, *p* = 0.000), ALT (I^2^ = 54.5%, *p* = 0.040), ALB (I^2^ = 79.5%, *p* = 0.000), and total bilirubin (TBIL) (I^2^ = 74.7%, *p* = 0.008) results due to their high heterogeneity. Decreased ALB levels (HR 0.91, 95% CI 0.86–0.96) was identified as an effective predictor for SFTS mortality, showing statistical significance (*p* < 0.05). In contrast, the other liver function indicators showed no statistical significance; thus, they could not yet be considered effective predictors for SFTS mortality (Figure S4).

#### 3.3.5. Association of Kidney Function Indicators with SFTS Mortality

We adopted a random-effects model for the blood urea nitrogen (BUN) (I^2^ = 92.4%, *p* = 0.000), creatinine (Cr) (I^2^ = 60.2%, *p* = 0.040), and uric acid (UA) (I^2^ = 66.3%, *p* = 0.051) results due to their high heterogeneity. None of these kidney function indicators were statistically significant (*p* > 0.05); thus, they could not yet serve as good predictors for SFTS mortality (Figure S5).

#### 3.3.6. Association of Myocardial Function Indicators with SFTS Mortality

Due to the great heterogeneity in the CK (I^2^ = 66.6%, *p* = 0.011), creatine kinase-myocardial band (CK-MB) (I^2^ = 59.7%, *p* = 0.083), and lactate dehydrogenase (LDH) (I^2^ = 76.6%, *p* = 0.000) results, a random-effects model was utilized. Elevation of the CK-MB levels (OR 1.01, 95% CI 1.01–1.02) was effective in predicting SFTS mortality, with statistical significance (*p* < 0.05). The other myocardial function indicators were not statistically significant; thus, they could not yet serve as good predictors for SFTS mortality (Figure S6).

#### 3.3.7. Association of Other Laboratory Parameters with SFTS Mortality

Since we observed great heterogeneity in the procalcitonin (PCT) (I^2^ = 86.8%, *p* = 0.000), CRP (I^2^ = 91.1%, *p* = 0.000), DD (I^2^ = 88.4%, *p* = 0.000), and potassium (K^+^) (I^2^ = 76.1%, *p* = 0.015) results, a random-effects model was utilized. The results showed that elevation of the PCT results (HR 1.27, 95% CI 1.10–1.47) could effectively predict SFTS mortality, with statistical significance (*p* < 0.05). The other parameters showed no statistical significance; thus, they could not yet act as good predictors for SFTS mortality (Figure S7).

### 3.4. Subgroup Analyses

We performed subgroup analyses by study site, study type, and country. In the one-center studies, levels viral load (OR 1.96, 95% CI 1.55–2.47), NLR (HR 1.21, 95% CI 1.14–1.27), LYM% (HR 0.96, 95% CI 0.94–0.98), NEU% (HR 1.02, 95% CI 1.01–1.04), PLT (HR 0.97, 95% CI 0.96–0.99), WBC (HR 1.18, 95% CI 1.03–1.36), ALB (HR 0.85, 95% CI 0.74–0.98), and PCT (HR 1.19, 95% CI 1.11–1.28) were identified as good predictors for SFTS mortality. In levels two-center studies, PLT (HR 0.98, 95% CI 0.96–0.99) was effective in predicting SFTS mortality. In levels retrospective studies, LYM% (HR 0.96, 95% CI 0.94–0.98), NEU% (HR 1.02, 95% CI 1.01–1.04), PLT (HR 0.98, 95% CI 0.97–0.99), WBC (HR 1.07, 95% CI 1.02–1.13), and ALB (HR 0.90, 95% CI 0.85–0.96) were effective predictors for SFTS mortality. In China, APTT (OR 1.06, 95% CI 1.04–1.09) was a good predictor for SFTS mortality. All of the above results displayed statistical significance (*p* < 0.05). In summary, the study site, study type, and country were not sources of heterogeneity. Some of the parameters with high heterogeneity underwent no subgroup analysis due to limited sample sizes or consistent characteristics (Figures S8–S12).

### 3.5. Sensitivity Analysis and Publication Bias

Leave-one-out sensitivity analyses were conducted, and the results revealed that except for two studies on TT, none of the studies had an unfavorable effect on the pooled effect size, validating the robustness of the results (Figures S13–S19). Additionally, we found significant publication bias in the viral load (*p* = 0.010), APPT (*p* = 0.033), PT (*p* = 0.010), CK (*p* = 0.020), and LDH (*p* = 0.001) results, which were then corrected for using the trim-and-fill method. The results had no great change before and after correction, suggesting that the publication bias was small and did not affect the reliability of the overall results (Figures S20–S26).

## 4. Discussion

The accurate identification of risk factors for disease progression is highly valuable for improving the clinical diagnosis and treatment and reducing the mortality risk of novel infectious diseases that lack effective treatment and vaccines. We conducted a meta-analysis of the association between SFTS and laboratory parameters and addressed key problems in previous studies, such as limited coverage of laboratory parameters, small sample sizes, and inconsistent conclusions for some parameters. We found that elevation of the viral load, NLR, NEU%, WBC, APTT, PT, CK-MB, and PCT and decreases in LYM%, PLT, and ALB corresponded to significantly increased SFTS mortality. Furthermore, the predictive value of the viral load, NLR, NEU%, LYM%, PLT, WBC, APTT, ALB, and PCT was validated by subgroup analyses, suggesting the close link of abnormalities of these parameters to the mortality risk.

Consistent with previous studies [42,54,55], a high viral load was considered an independent risk factor for SFTS mortality, which had a positive correlation with an unfavorable prognosis. High viral loads significantly upregulate macrophage inflammatory protein-1α and IFN-inducible protein-10, simultaneously inhibit the release of secretory factors from activated T cells [56], and trigger the overexpression of pro-inflammatory factors (e.g., IL-6, IL-10, TNF-α). As a result, a “cytokine storm” is induced, and worse systemic inflammation and multi-organ injury ultimately occur [57]. Therefore, the association between high viral loads and increased SFTS mortality underscores the need for future research and development of effective anti-viral replication and anti-inflammation therapies.

No consensus has been reached on the predictive capacity of blood routine indicators in previous studies, possibly because of the variations in sample sizes and statistical indicators. We meta-analyzed the latest evidence and found that elevated NLR was a predictor for SFTS mortality, consistent with the findings of Wei [53] and Wang [58], whose mechanism is closely related to direct viral immunosuppression, inflammatory imbalance, and multi-organ injury. Decreased PLT levels had a great correlation with the mortality risk, consistent with the study by Wang [16], which may be related to vascular endothelial dysfunction resulting from arginine deficiency in SFTS patients [59] and viral infections directly suppressing bone marrow hematopoiesis or promoting the clearance of virally adherent platelets by splenic macrophages [60]. Decreased WBC levels is a typical feature of SFTS [12], although its association with the prognosis remains controversial. Liu [32] and Wang [37] confirmed no significant correlation between WBC abnormalities and the mortality risk. In this study, elevated WBC levels was identified as a predictor for mortality risk, consistent with the results of Liu’s study [30]. It is hypothesized that elevated WBC levels may reflect the excessive inflammatory response to the virus and secondary bacterial infections, thereby raising the mortality risk.

This study showed that elevated APTT levels was associated with SFTS mortality, consistent with the finding of Wang’s study [61], which verified the prognostic value of PT, similar to the study by Wang [16]. Therefore, early clinical monitoring of coagulation function indicators is crucial for prognostic assessments, although the pathologic mechanism of coagulation dysfunction as a common complication of SFTS has not been fully clarified. As suggested by the available evidence, endothelial dysfunction, thrombocytopenia, and an imbalance of endogenous and exogenous coagulation pathways may be implicated in coagulation disorders [62]. In addition, acute liver injury triggered by SFTSV infections can lead to decreased synthesis of coagulation factors [57], inducing secondary coagulation disorders caused by disseminated intravascular coagulation.

SFTS is a multi-system complex disease, and liver, kidney, and myocardial function parameters are determined as key predictors for fatal outcomes of SFTS [63,64]. Several studies have pointed out that abnormalities of liver serologic parameters in SFTS patients may indirectly reflect hepatocellular injury and correlate with coagulation disorders [16,65]. The early studies on the prognostic value of ALB have not reached a unified conclusion due to limitations of sample sizes and variations in study populations. In this study, decreased ALB levels was identified as a risk factor for SFTS mortality, possibly because SFTSV infections restrain the ALB-synthesizing capacity of the liver via systemic inflammatory responses, and inflammation-mediated vascular endothelial injury leads to ALB leakage into the tissue space [32]. However, previous studies have shown that acute liver injury should also present with elevated AST and ALT levels [32,47], and their increased levels could significantly increase the risk of SFTS mortality [16], although the two did not show statistical significance in this study, which may be attributed to the high heterogeneity in the included studies and the limited number of included studies. In addition, the kidney is a potential target organ of SFTSV; severe kidney injury (e.g., elevation of BUN, Cr, and UA levels) is associated with the mortality risk, which has been confirmed by Gui [66], Wang [58] and Wang [67]. Wang [16] also proved that elevated BUN and Cr levels are linked to a poor prognosis. However, no statistical significance was detected for BUN, Cr, and UA in this study, possibly attributed to the number and heterogeneity of the included studies. Additionally, myocardial injury, a common complication of SFTS, can significantly raise the risk of critical illness [44]. CK has been recognized as an independent predictor for SFTS mortality [32,68], while this study suggested that elevated CK-MB significantly increased the mortality risk, consistent with the findings of Wang’s study [16]. It is currently believed that elevated CK levels may be associated with virus- or inflammation-mediated rhabdomyolysis, myocardial injury, and multi-organ failure [69], although in-depth exploration is still required for the specific mechanism of the predictive efficiency of CK-MB.

Wang [16] pointed out a strong correlation of elevated CRP, DD, and K levels with SFTS mortality. This study also suggested the potential predictive value of the three parameters in the mortality risk, although the results were not statistically significant, possibly due to high heterogeneity in the included studies; therefore, the clinical reference value of the findings was limited. Notably, PCT can serve as a key biomarker for guiding antibiotic treatment for severe sepsis [70]. Wang [61] argued that PCT is a risk factor for SFTS mortality, further evidenced by this study. In which elevated PCT levels greatly raised the SFTS mortality. The possible mechanism involves a combination of direct stimulation of PCT secretion due to an SFTSV infection-induced cytokine storm, further increase in PCT production due to secondary bacterial infection, and impaired metabolic clearance due to liver and kidney injury.

This study covered a larger sample size and more laboratory parameters, with HR or OR as the effect size, to more clearly clarify the predictive efficiency of clinical laboratory parameters for SFTS mortality. However, some limitations are worth noting. First, significant heterogeneity was present for some parameters, and the included original studies on TT had a risk of bias. Second, some parameters were described in only one study in the subgroup analysis, restricting the explanation of heterogeneity. Third, the available data were mostly from East Asia (especially China), and multicenter data were lacking. Fourth, only English studies were included and high-quality evidence in other languages was not covered, and the methodological differences may potentially affect the robustness of the findings. Fifth, some confounding factors, such as the older age of the subjects and possible underlying comorbidities, were not incorporated in the analysis, which may have had some impact on the study results.

## 5. Conclusions

This study covered large samples and more objective laboratory parameters to break through the limitations of traditional clinical assessments, with HR or OR as the key effect size. The statistical framework was optimized by the strict methodological standards. In summary, this study contributes to the growing body of evidence elucidating the impact of laboratory parameters on mortality risk in SFTS patients. We found that abnormal levels of viral load, blood routine, coagulation function, liver function, and myocardial function indicators significantly increased the risk of SFTS mortality, especially the viral load, NLR, NEU%, LYM%, WBC, PLT, APTT, PT, ALB, CK-MB, and PCT. The findings are of great value in guiding the development of early clinical interventions in China and other SFTS-endemic regions, and offer a scientific basis for reducing mortality and ameliorating outcomes. In the future, data from studies of multiple languages and countries should be obtained to enrich the evidence and further validate the clinical prognostic value of parameters with high heterogeneity.

## Figures and Tables

**Table 1:** Baseline characteristics of the 33 observational studies included in the systematic review and meta-analysis.

Author and Year	Study Type	Study Design	Country	Study Sites	Hospital Level	N (M/F)	Fatal Number	Non-Fatal Number	Mean Age (Years)	Laboratory Parameters	NOS
Cao et al., 2024 [21]	Retrospective	Cohort study	China	Two	Tertiary	217 (92/125)	60	157	64.0	LYM MONO PLT ALB ALP ALT GGT BUN CK CRP PCT PT	High
Fang et al., 2024 [22]	Retrospective	Case control study	China	One	Tertiary	394 (189/205)	92	302	65.68 ± 10.14	PLT ALT AST CK CK-MB LDH BUN Cr PT APTT TT DD FIB	High
Guo et al., 2024 [23]	Retrospective	Cohort study	China	One	Tertiary	610 (352/258)	81	529	61.47	CK-MB PT	High
Hou et al., 2024 [24]	Retrospective	Cohort study	China	One	Tertiary	93 (38/55)	37	56	63.94 ± 2.63	APTT LDH PLT ALB PCT TT DD	High
Huang et al., 2023 [25]	Retrospective	Cohort study	China	One	Tertiary	47 (28/19)	23	24	62.61 ± 5.03	LDH PCT APTT TT	High
Kim et al., 2023 [26]	Retrospective	Cohort study	Republic of Korea	One	Tertiary	91 (53/38)	10	81	60.86 ± 15.01	APTT	High
Li et al., 2024 [27]	Prospective	Cohort study	China	Multi	Secondary/Tertiary	686 (330/356)	87	599	65.99 ± 2.41	AST LDH CK CK-MB	High
Li et al., 2023 [28]	Retrospective	Cohort study	China	One	Tertiary	200 (94/106)	35	165	70.76 ± 3.11	WBC NEU LYM MONO PLT ALT APTT AST CRP CK CK-MB GGT LDH BUN PCT PT Cr UA DD ALB Viral load	High
Liang et al., 2024 [29]	Retrospective	Cohort study	China	One	Tertiary	580 (242/338)	111	469	60.98 ± 2.44	PLT Viral load Cr	High
Liu et al., 2022 [30]	Retrospective	Cohort study	China	One	Tertiary	182 (88/94)	24	158	59.64 ± 12.74	NEU LYM NLR WBC	High
Liu et al., 2022 [31]	Retrospective	Case control study	China	One	Tertiary	194 (101/93)	23	171	62.39 ± 11.85	AST	High
Liu et al., 2022 [32]	Retrospective	Cohort study	China	One	Tertiary	155 (77/78)	22	133	61.98 ± 11.70	ALB ALP ALT APTT AST Cr CRP GGT Hb LDH LYM LYM% MONO NEU NEU% PCT PLT PT RBC TBIL TT WBC	High
Peng et al., 2024 [33]	Retrospective	Cohort study	China	Multi	Secondary/Tertiary	541 (275/266)	60	481	62.02 ± 2.46	Cr APTT AST	High
Qian et al., 2023 [34]	Retrospective	Cohort study	China	Multi	Secondary/Tertiary	882 (428/454)	157	725	63.99 ± 2.35	CK APTT AST	High
Wang et al., 2022 [35]	Retrospective	Cohort study	China	Two	Tertiary	122 (64/58)	20	102	61.66 ± 13.02	APTT	High
Wang et al., 2020 [36]	Retrospective	Cohort study	China	One	Tertiary	51 (27/24)	16	35	57.52 ± 12.37	WBC NEU LYM Hb PLT ALT AST CK LDH BUN Cr PT APTT FIB	High
Wang et al., 2019 [37]	Prospective	Cohort study	China	Multi	Secondary/Tertiary	429 (228/201)	69	360	60.8 ± 12.1	WBC NEU NEU% LYM LYM% MONO RBC Hb PLT LDH CK-MB BUN Cr PT APTT ALT ALB	High
Wang et al., 2024 [38]	Retrospective	Cohort study	China	One	Tertiary	214 (95/119)	57	157	67.9 ± 10.6	LYM% NEU	High
Wang et al., 2024 [39]	Retrospective	Cohort study	China	Two	Tertiary	437 (190/247)	101	336	NA	WBC NEU LYM MONO PLT RBC Hb CRP PCT ALT GGT TBIL ALB ALP BUN Cr PT APTT TT	High
Wei et al., 2022 [53]	Retrospective	Cohort study	China	Two	Tertiary	228 (107/121)	51	177	62.96 ± 3.02	NLR BUN NEU LYM PLT ALP ALB	High
Xia et al., 2023 [40]	Retrospective	Cohort study	China	Multi	Tertiary	161 (63/98)	26	135	64.22 ± 3.23	WBC PLT ALT CK Hb Viral load	High
Xiao et al., 2024 [41]	Retrospective	Cohort study	China	One	Tertiary	372 (166/206)	79	293	66.93 ± 2.56	Viral load PT	High
Xiong et al., 2016 [42]	Retrospective	Cohort Study	China	One	Tertiary	179 (71/108)	34	145	58.08 ± 11.86	Viral load PLT NEU% LYM% ALT AST GGT Cr LDH CK TT APTT	High
Yang et al., 2017 [43]	Retrospective	Cohort Study	China	One	Tertiary	123 (62/61)	31	92	59.50 ± 11.52	WBC PLT Cr K^+^ LDH APTT CK ALB	High
Yang et al., 2023 [44]	Retrospective	Cohort Study	China	One	Tertiary	109 (50/59)	27	82	67.64 ± 3.75	LYM ALB PLT DD	High
Yang et al., 2024 [45]	Retrospective	Case control study	China	One	Tertiary	292 (150/142)	72	220	67.84 ± 2.45	DD	High
Zhang et al., 2024 [46]	Retrospective	Cohort study	China	Multi	Tertiary	304 (155/149)	70	234	61.95 ± 3.14	WBC PLT ALT LDH ALB Cr PT	High
Zhang et al., 2024 [48]	Retrospective	Cohort study	China	Multi	Tertiary	292 (150/142)	69	223	61.45 ± 3.27	WBC RBC Hb PLT ALT GGT Cr BUN TBIL PT APTT	High
Zhang et al., 2024 [47]	Retrospective	Cohort study	China	One	Tertiary	291 (151/140)	65	226	62.92 ± 3.33	WBC PLT ALT ALP GGT TBIL Cr PT	High
Zhang et al., 2025 [49]	Retrospective	Cohort study	China	One	Tertiary	101 (55/46)	17	84	65.25 ± 10.67	NEU% LYM% NLR PLT AST BUN LDH TT APTT	High
Zhang et al., 2023 [50]	Retrospective	Cohort study	China	One	Tertiary	208 (110/98)	37	171	65 ± 8	WBC NEU NEU% LYM LYM% Hb PLT ALT AST TBIL ALB ALP GGT LDH BUN K^+^ CK-MB PT APTT TT FIB DD CRP PCT Viral load	High
Zhang et al., 2024 [51]	Retrospective	Cohort study	China	One	Tertiary	290 (147/143)	50	240	64.34 ± 7.21	Cr	High
Zhong et al., 2024 [52]	Retrospective	Cohort study	China	One	Tertiary	427 (189/238)	86	341	66.93 ± 2.44	LYM MONO NEU PLT ALB UA K^+^ ALT AST LDH TT PT APTT FIB Cr Viral load CK	High

LYM, lymphocyte; LYM%, lymphocyte percentage; NEU, neutrophil; NEU%, neutrophil percentage; MONO, monocyte; WBC, white blood cell; RBC, red blood cell; Hb, hemoglobin; NLR, neutrophil-to-lymphocyte ratio; PLT, platelet; CK, creatine kinase; CK-MB, creatine kinase–myocardial band; LDH, lactate dehydrogenase; AST, aspartate aminotransferase; ALT, alanine aminotransferase; TBIL, total bilirubin; ALP, alkaline phosphatase; GGT, gamma-glutamyl transferase; ALB, albumin; Cr, creatinine; BUN, blood urea nitrogen; UA, uric acid; APTT, activated partial thromboplastin time; PT, prothrombin time; TT, thrombin time; FIB, fibrinogen; PCT, procalcitonin; CRP, C-reactive protein; DD, D-dimer; K^+^, potassium.

## Data Availability

The original contributions presented in this study are included in the article. Further inquiries can be directed to the corresponding authors.

## Supplementary Materials

The following supporting information can be downloaded at: https://www.mdpi.com/article/10.3390/tropicalmed10070193/s1, Table S1. PRISMA 2020 checklist [71]. Table S2. Search strategy. Table S3. Newcastle–Ottawa Quality Assessment Scale of the 30 cohort studies. Table S4. Newcastle–Ottawa Quality Assessment Scale of the 3 case control studies. Figure S1. Forest plot of viral load for predicting the risk of mortality in severe fever with thrombocytopenia syndrome. OR, odds ratio; CI, confidence interval. NOTE: I^2^ > 50%, weights are from a random-effects analysis. Figure S2. Forest plot of blood routine indicators for predicting the risk of mortality in severe fever with thrombocytopenia syndrome. HR, hazard ratio; CI, confidence interval. NOTE: I^2^ > 50%, weights are from a random-effects analysis. Figure S3. Forest plot of coagulation indicators for predicting the risk of mortality in severe fever with thrombocytopenia syndrome. OR, odds ratio; CI, confidence interval. NOTE: I^2^ > 50%, weights are from a random-effects analysis. Figure S4. Forest plot of liver function indicators for predicting the risk of mortality in severe fever with thrombocytopenia syndrome. HR, hazard ratio; CI, confidence interval. NOTE: I^2^ > 50%, weights are from a random-effects analysis. Figure S5. Forest plot of renal function indicators for predicting the risk of mortality in severe fever with thrombocytopenia syndrome. OR, odds ratio; CI, confidence interval. NOTE: I^2^ > 50%, weights are from a random-effects analysis. Figure S6. Forest plot of myocardial function indicators for predicting the risk of mortality in severe fever with thrombocytopenia syndrome. OR, odds ratio; CI, confidence interval. NOTE: I^2^ > 50%, weights are from a random-effects analysis. Figure S7. Forest plot of other laboratory parameters for predicting the risk of mortality in severe fever with thrombocytopenia syndrome. HR, hazard ratio; OR, odds ratio; CI, confidence interval. NOTE: I^2^ > 50%, weights are from a random-effects analysis. Figure S8. Subgroup analysis of viral load for predicting the risk of mortality in severe fever with thrombocytopenia syndrome. OR, odds ratio; CI, confidence interval. NOTE: I^2^ > 50%, weights are from a random-effects analysis. Figure S9. A subgroup analysis of blood routine indicators for predicting the risk of mortality in severe fever with thrombocytopenia syndrome. HR, hazard ratio; CI, confidence interval. NOTE: I^2^ > 50%, weights are from a random-effects analysis. Figure S10. A subgroup analysis of activated partial thromboplastin time for predicting the risk of mortality in severe fever with thrombocytopenia syndrome. OR, odds ratio; CI, confidence interval. NOTE: I^2^ > 50%, weights are from a random-effects analysis. Figure S11. A subgroup analysis of liver function indicators for predicting the risk of mortality in severe fever with thrombocytopenia syndrome. HR, hazard ratio; CI, confidence interval. NOTE: I^2^ > 50%, weights are from a random-effects analysis. Figure S12. A subgroup analysis of other laboratory parameters for predicting the risk of mortality in severe fever with thrombocytopenia syndrome. HR, hazard ratio; CI, confidence interval. NOTE: I^2^ > 50%, weights are from a random-effects analysis. Figure S13. A sensitivity analysis for the literature related to viral load. Figure S14. A sensitivity analysis for the literature related to blood routine indicators. Figure S15. A sensitivity analysis for the literature related to coagulation indicators. Figure S16. A sensitivity analysis for the literature related to liver function indicators. Figure S17. A sensitivity analysis for the literature related to renal function indicators. Figure S18. A sensitivity analysis for the literature related to myocardial function indicators. Figure S19. A sensitivity analysis for the literature related to other laboratory parameters. Figure S20. Publication bias assessment for viral load. Figure S21. Publication bias assessment for blood routine indicators. Figure S22. Publication bias assessment for coagulation indicators. Figure S23. Publication bias assessment for liver function indicators. Figure S24. Publication bias assessment for renal function indicators. Figure S25. Publication bias assessment for myocardial function indicators. Figure S26. Publication bias assessment for other laboratory parameters.
