# Supplementary material for: Prediction of Mortality by Clinical Laboratory Parameters in Severe Fever with Thrombocytopenia Syndrome: A Meta-Analysis
Source: Trop Med Infect Dis. 2025 Jul 9;10(7):193. doi: 10.3390/tropicalmed10070193 (PMC12300845; doi:10.3390/tropicalmed10070193)
Supplement: Supplementary file 1 [file tropicalmed-10-00193-s001.zip › Supplementary Figures.pdf]

Supplementary Figures

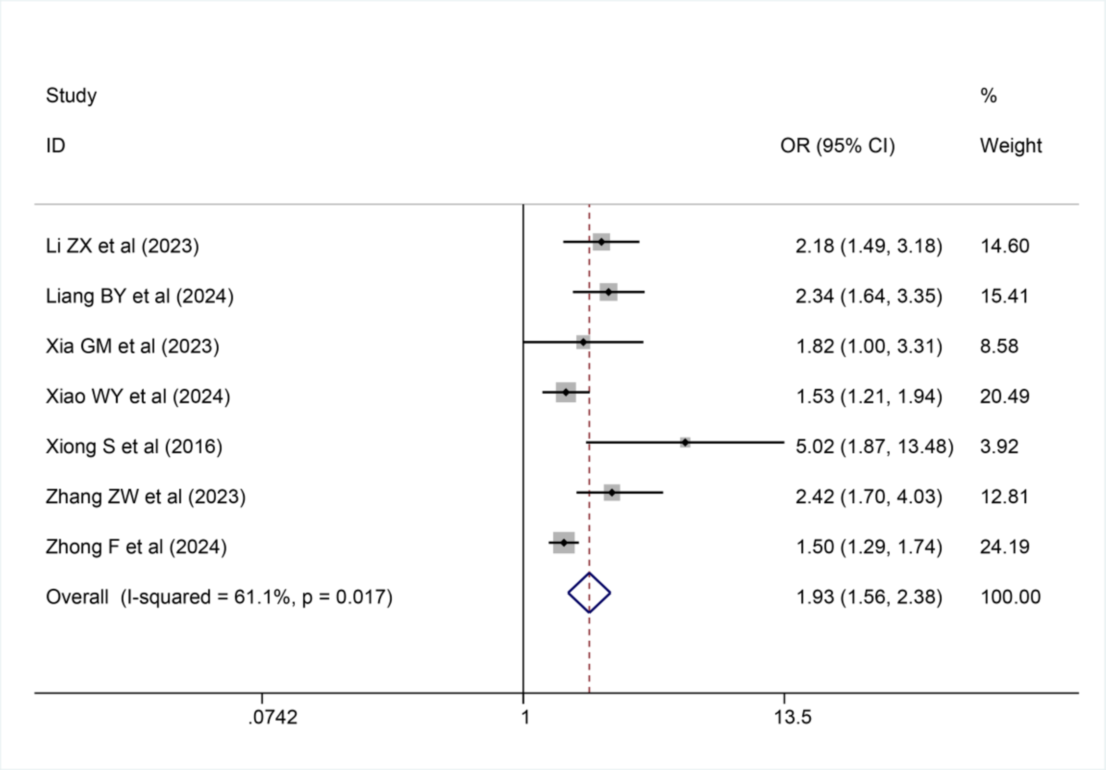

Figure S1. Forest plot of viral load for predicting the risk of mortality in severe fever with thrombocytopenia syndrome. OR, odds ratio; CI, confidence interval. NOTE: $I^2 > 50\%$, weights are from a random-effects analysis.

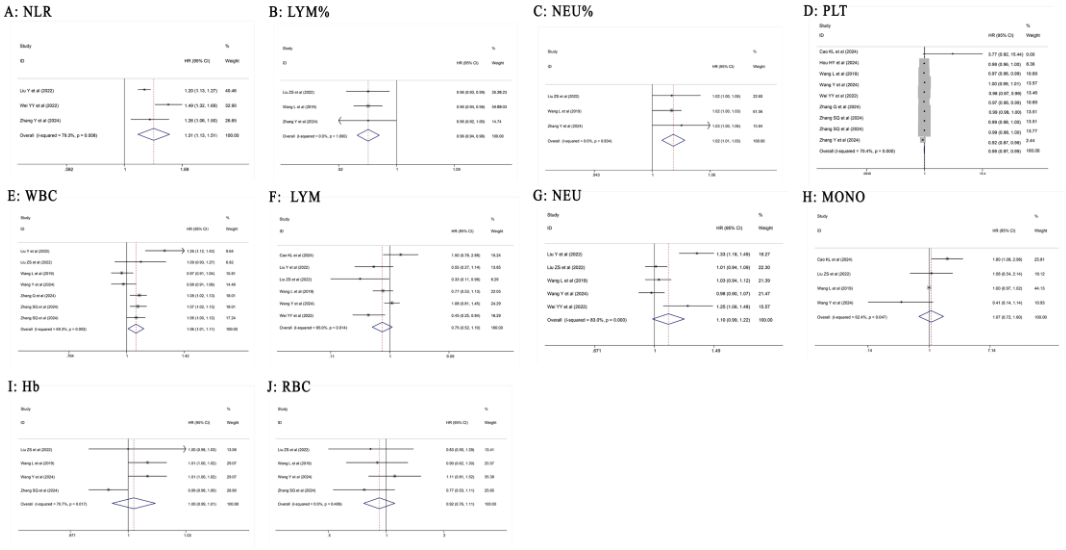

Figure S2. Forest plot of blood routine indicators for predicting the risk of mortality in severe fever with thrombocytopenia syndrome. HR, hazard ratio; CI, confidence interval. NOTE: $I^2 > 50\%$, weights are from a random-effects analysis.

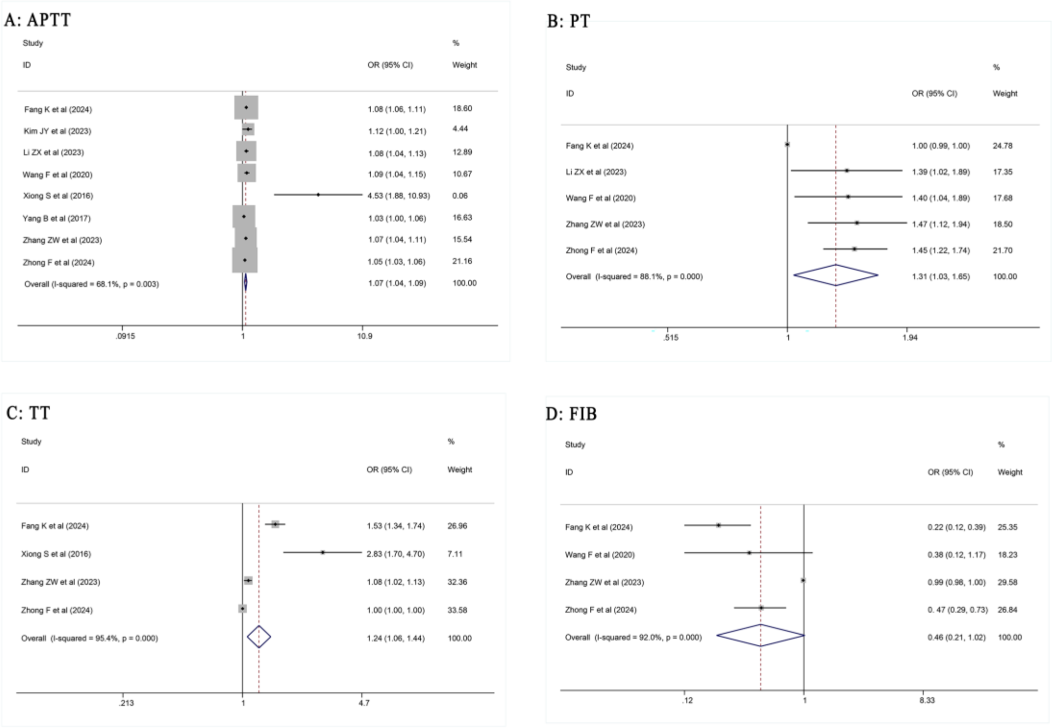

Figure S3. Forest plot of coagulation indicators for predicting the risk of mortality in severe fever with thrombocytopenia syndrome. OR, odds ratio; CI, confidence interval. NOTE: $I^2 > 50\%$, weights are from a random-effects analysis.

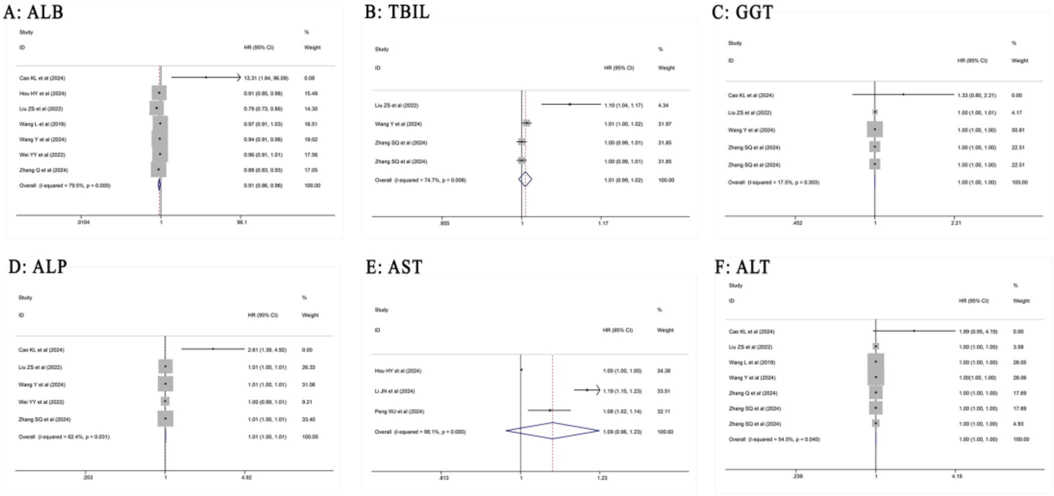

Figure S4. Forest plot of liver function indicators for predicting the risk of mortality in severe fever with thrombocytopenia syndrome. HR, hazard ratio; CI, confidence interval. NOTE: $I^2 > 50\%$, weights are from a random-effects analysis.

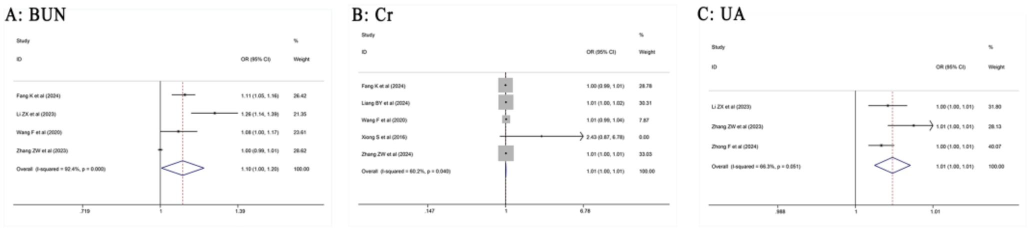

Figure S5. Forest plot of renal function indicators for predicting the risk of mortality in severe fever with thrombocytopenia syndrome. OR, odds ratio; CI, confidence interval. NOTE: $I^2 > 50\%$, weights are from a random-effects analysis.

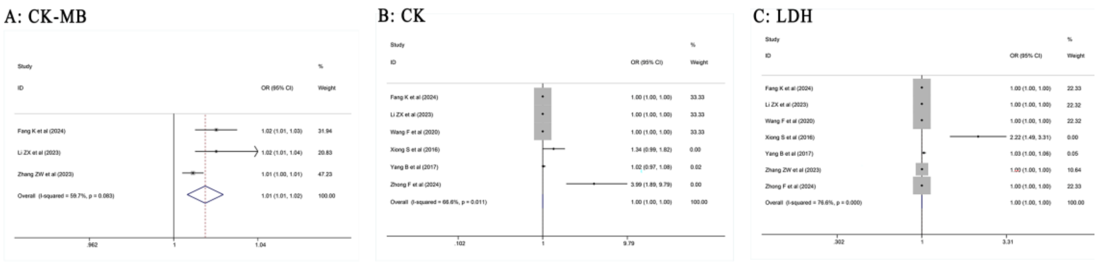

Figure S6. Forest plot of myocardial function indicators for predicting the risk of

mortality in severe fever with thrombocytopenia syndrome. OR, odds ratio; CI, confidence interval. NOTE: $I^2 > 50\%$, weights are from a random-effects analysis.

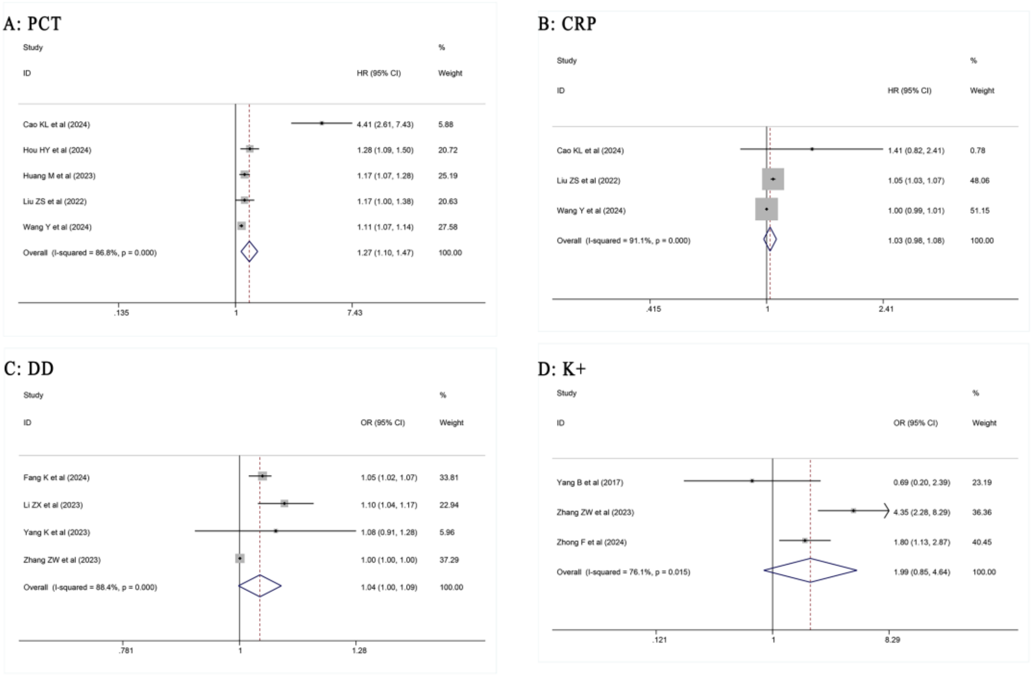

Figure S7. Forest plot of other laboratory parameters for predicting the risk of mortality in severe fever with thrombocytopenia syndrome. HR, hazard ratio; OR, odds ratio; CI, confidence interval. NOTE: $I^2 > 50\%$, weights are from a random-effects analysis.

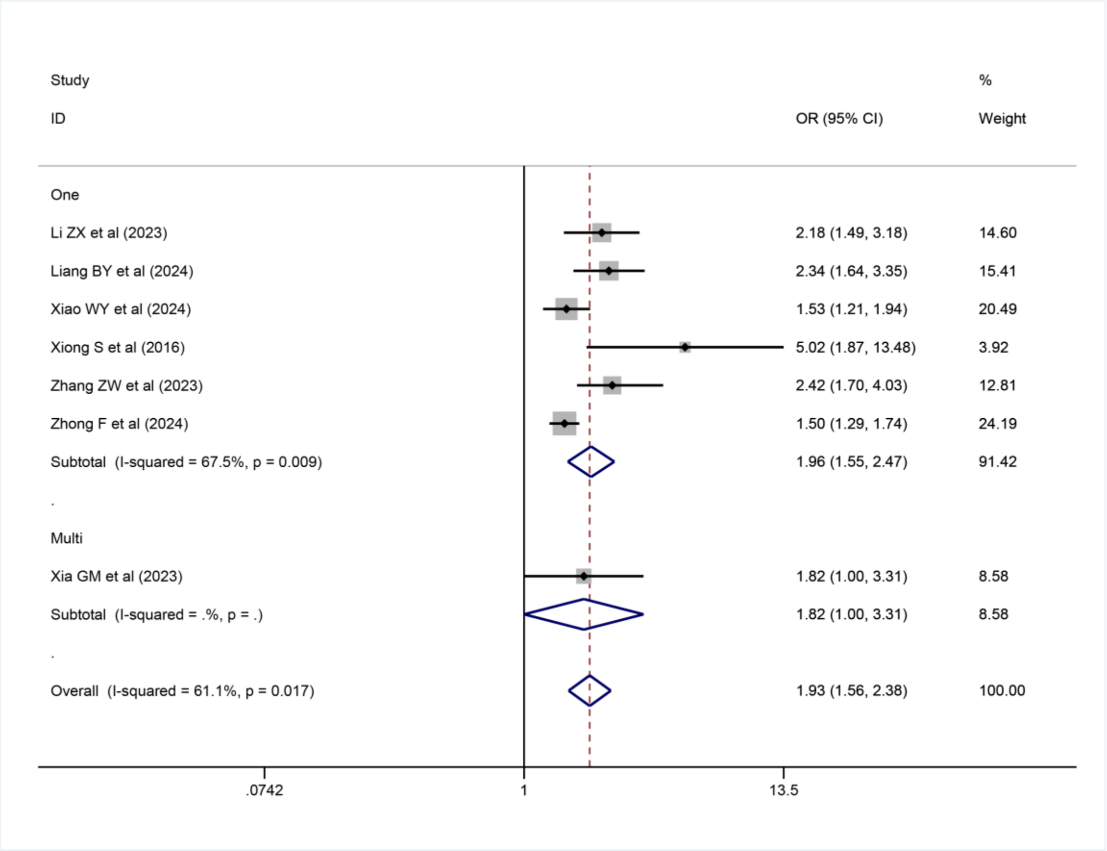

Figure S8. **Subgroup analysis of viral load for predicting the risk of mortality in severe fever with thrombocytopenia syndrome.** OR, odds ratio; CI, confidence interval. NOTE: $I^2 > 50\%$, weights are from a random-effects analysis.

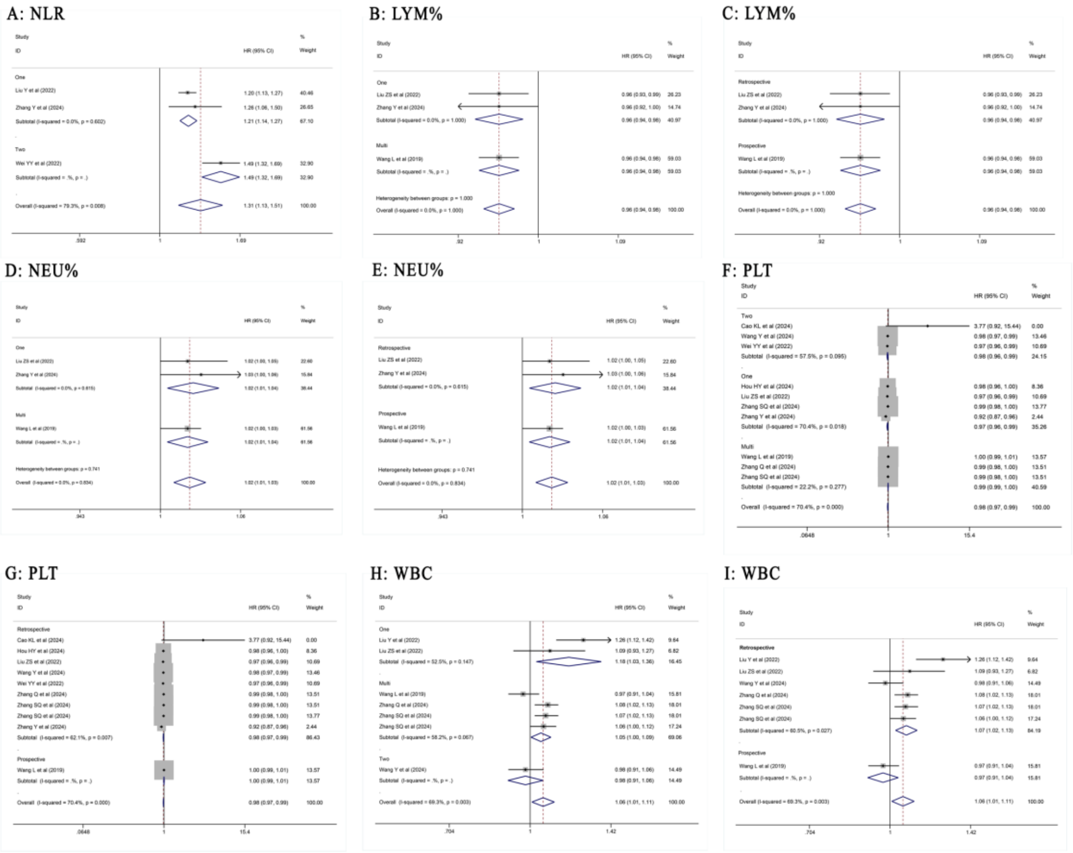
A: NLR
B: LYM%
C: LYM%
D: NEU%
E: NEU%
F: PLT
G: PLT
H: WBC
I: WBC

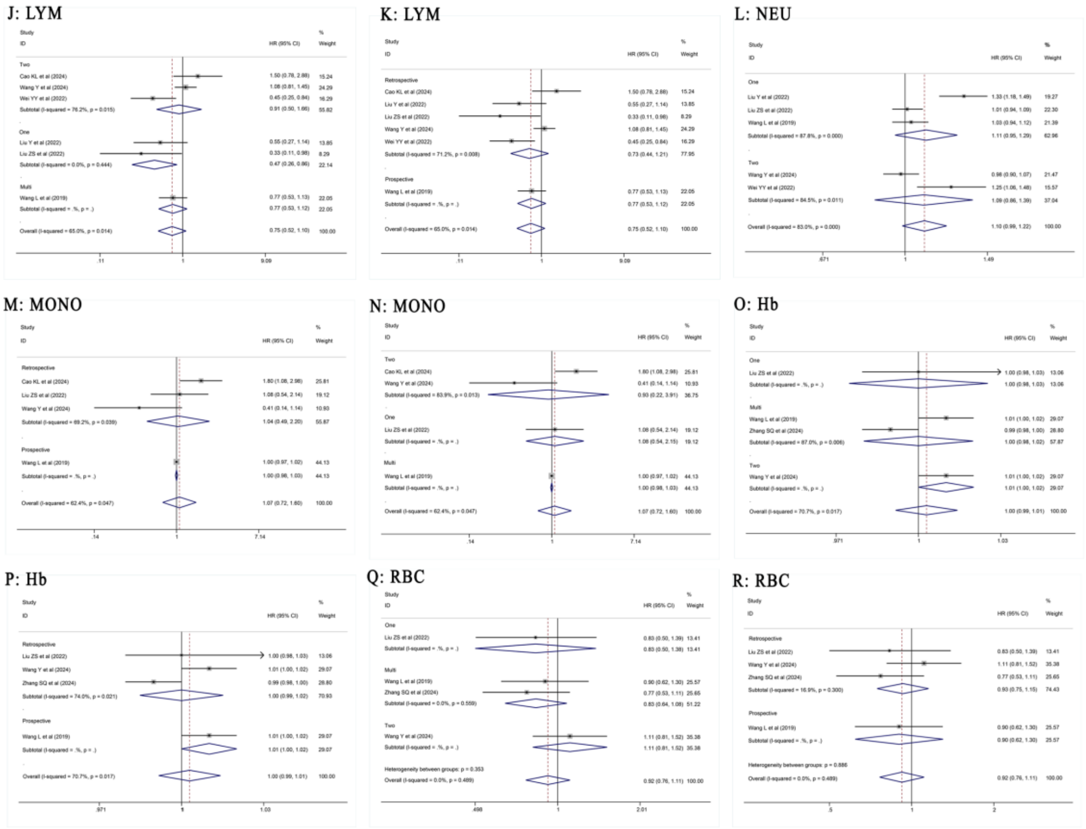

Figure S9. Subgroup analysis of blood routine indicators for predicting the risk of mortality in severe fever with thrombocytopenia syndrome. HR, hazard ratio; CI, confidence interval. NOTE: $I^2 > 50\%$, weights are from a random-effects analysis.

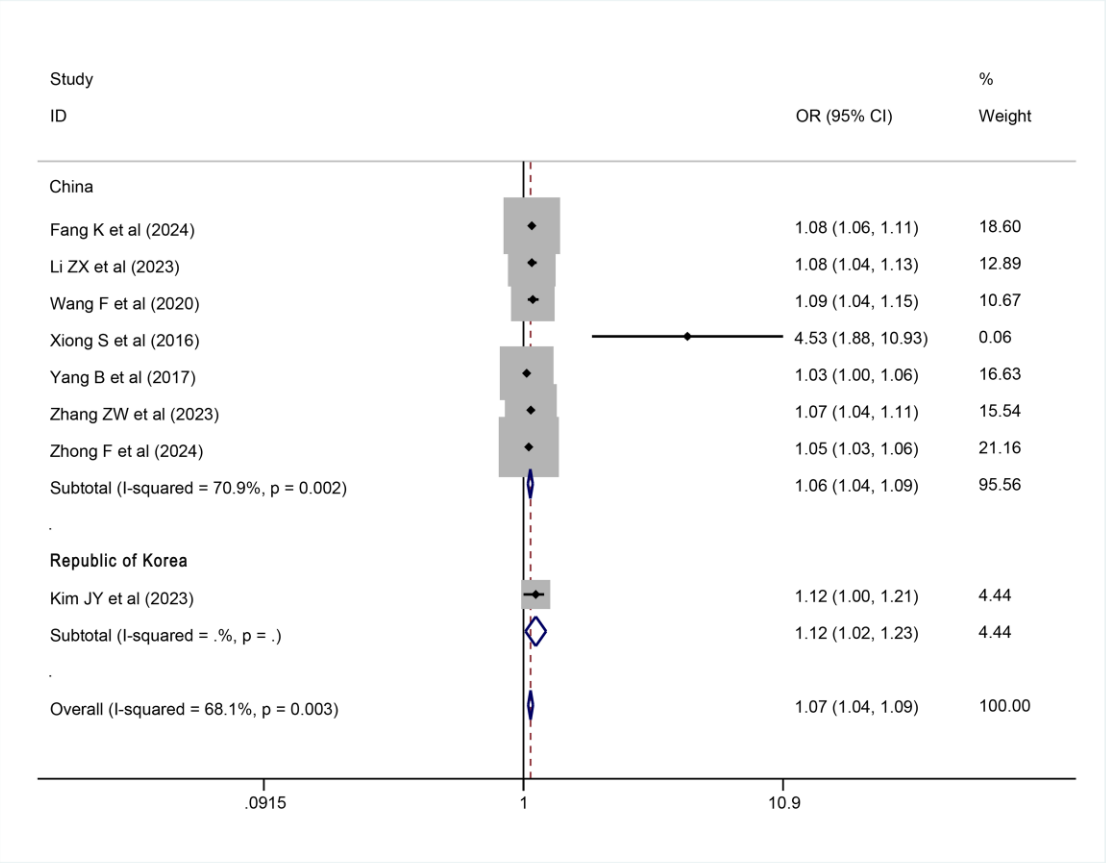

Figure S10. Subgroup analysis of activated partial thromboplastin time for predicting the risk of mortality in severe fever with thrombocytopenia syndrome. OR, odds ratio; CI, confidence interval. NOTE: I^2 > 50%, weights are from a random-effects analysis.

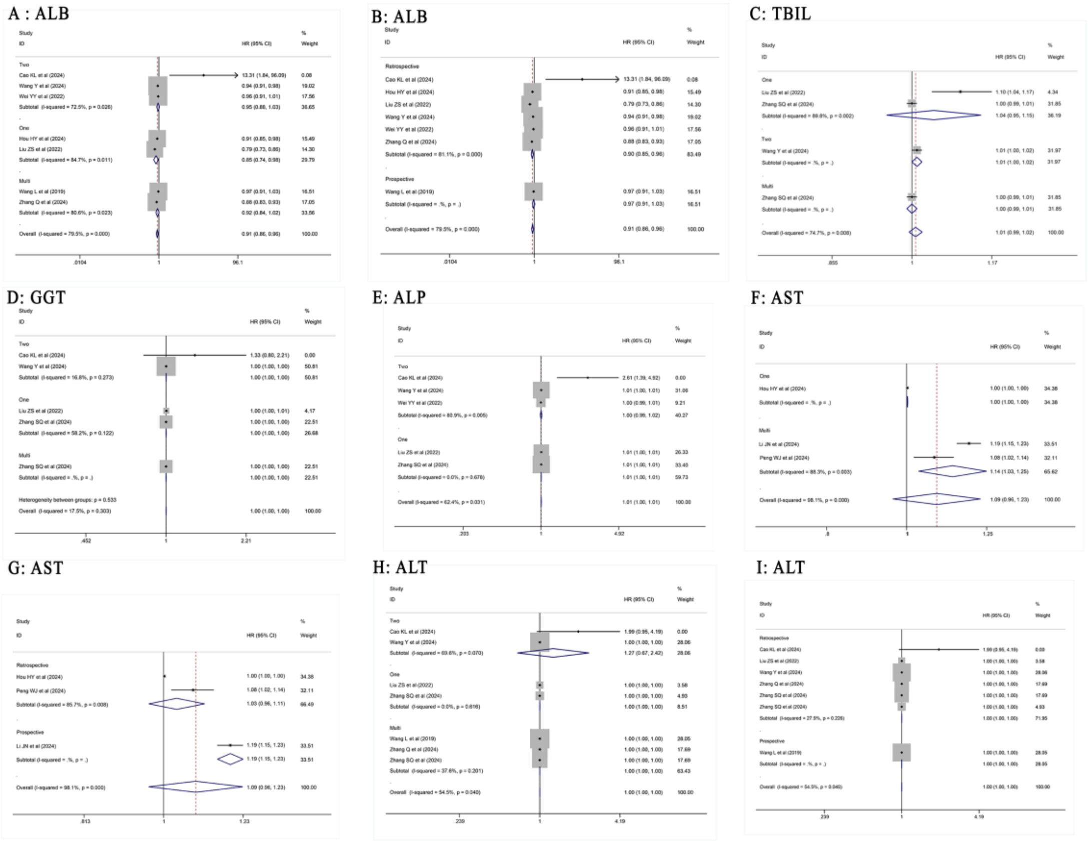

Figure S11. Subgroup analysis of liver function indicators for predicting the risk of mortality in severe fever with thrombocytopenia syndrome. HR, hazard ratio; CI, confidence interval. NOTE: $I^2 > 50\%$, weights are from a random-effects analysis.

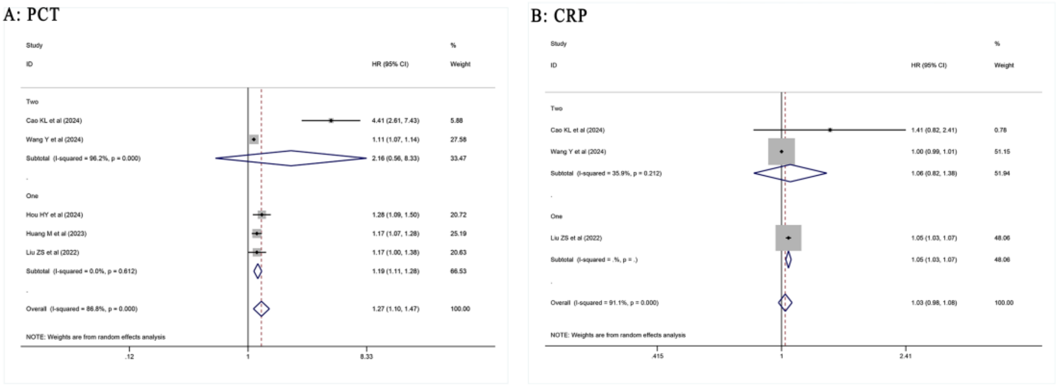

Figure S12. Subgroup analysis of other laboratory parameters for predicting the risk of mortality in severe fever with thrombocytopenia syndrome. HR, hazard ratio; CI, confidence interval. NOTE: $I^2 > 50\%$, weights are from a random-effects

analysis.

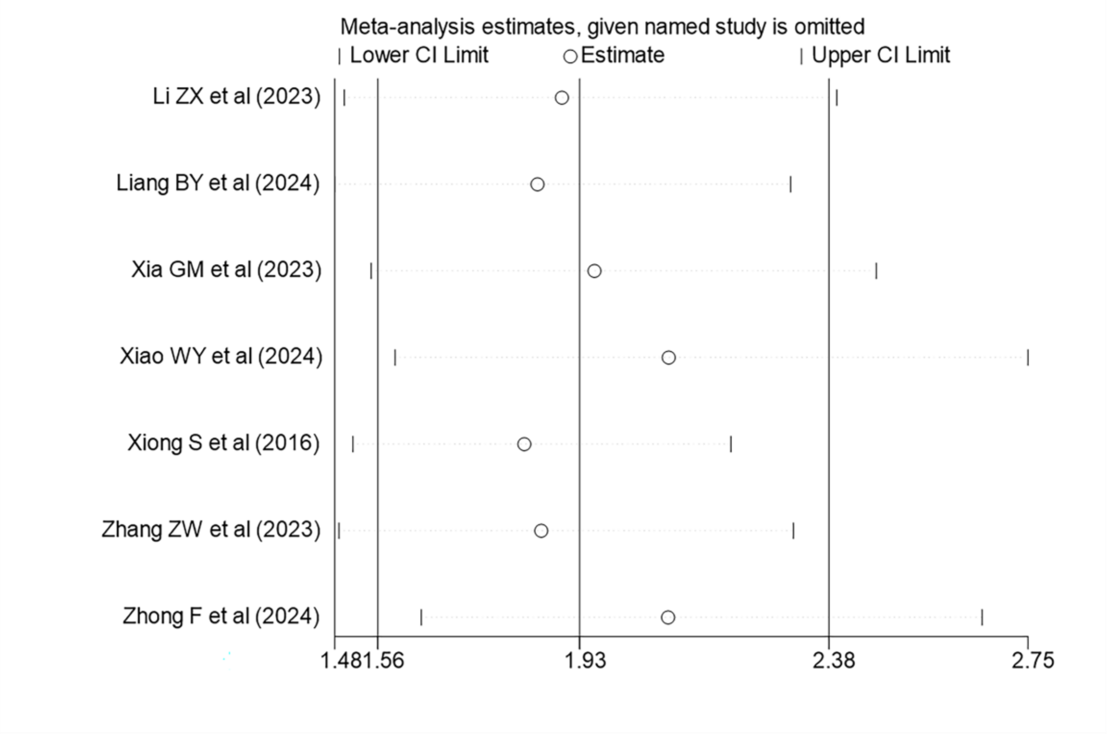

Figure S13. A sensitivity analysis for the literature related to viral load.

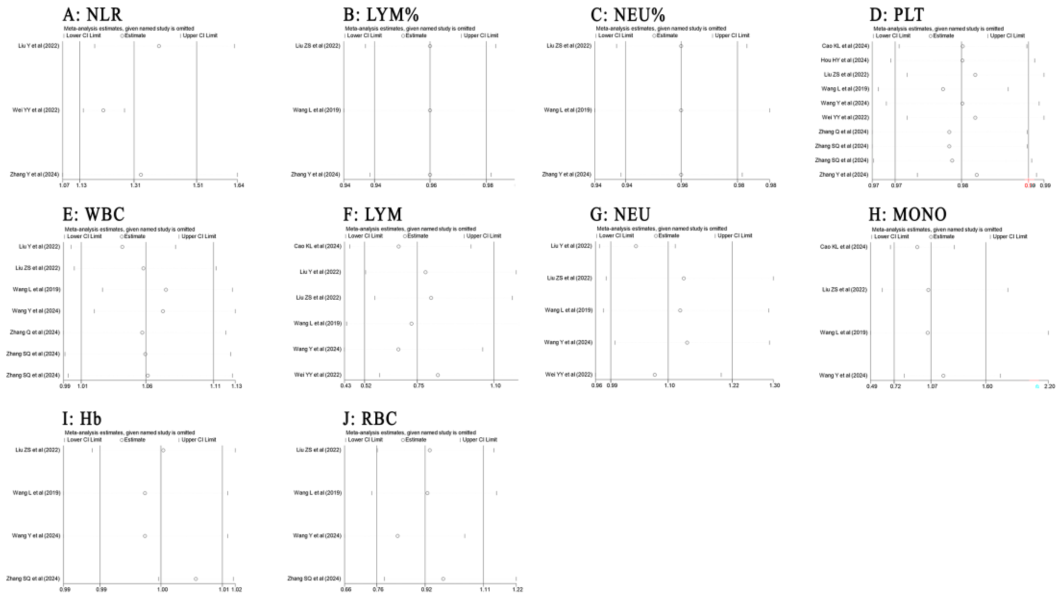

Figure S14. A sensitivity analysis for the literature related to blood routine indicators.

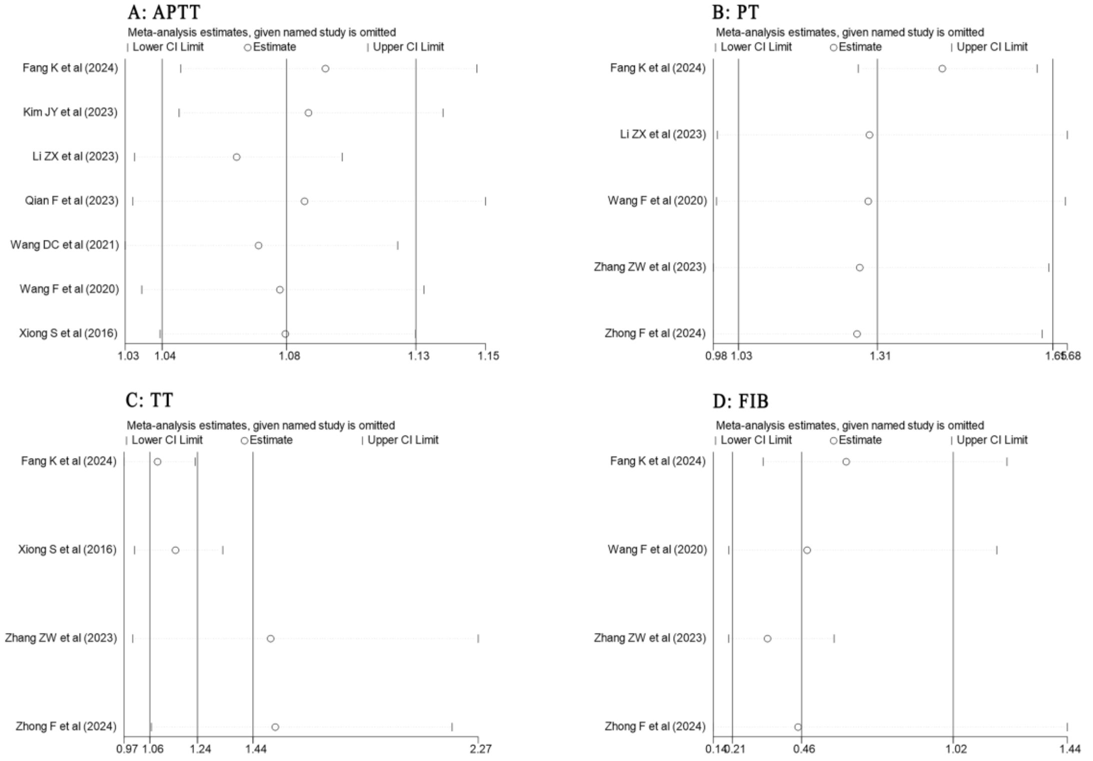

Figure S15. A sensitivity analysis for the literature related to coagulation indicators.

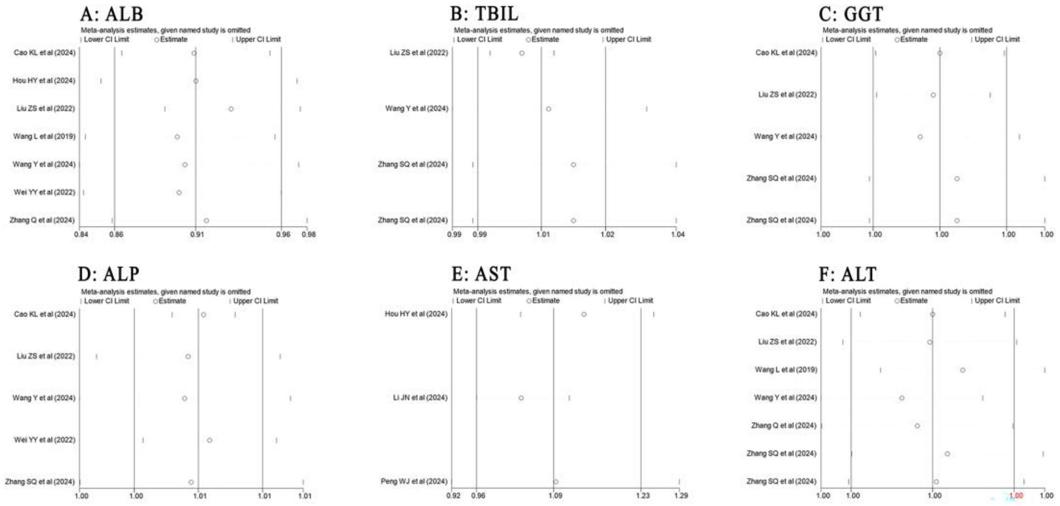

Figure S16. A sensitivity analysis for the literature related to liver function indicators.

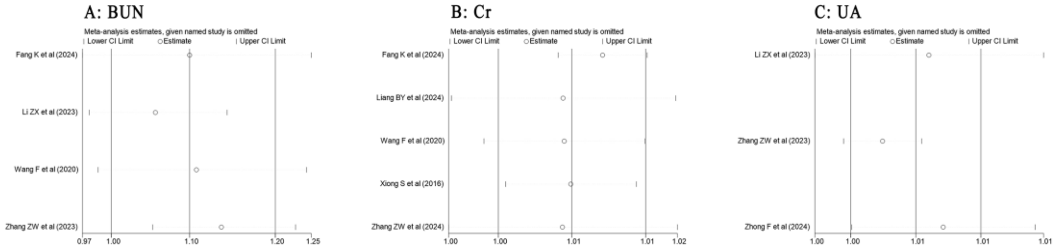

Figure S17. A sensitivity analysis for the literature related to renal function indicators.

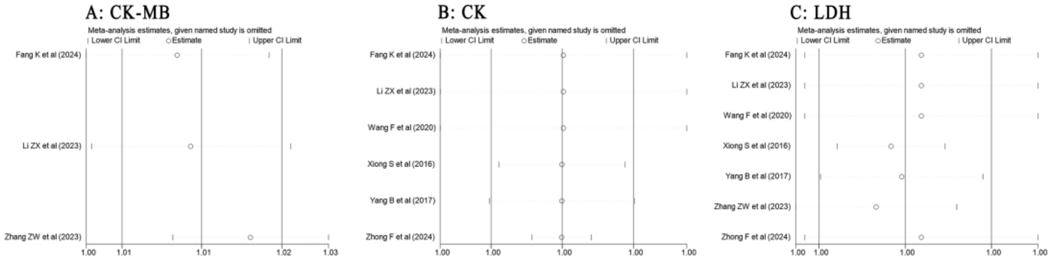

Figure S18. A sensitivity analysis for the literature related to myocardial function indicators.

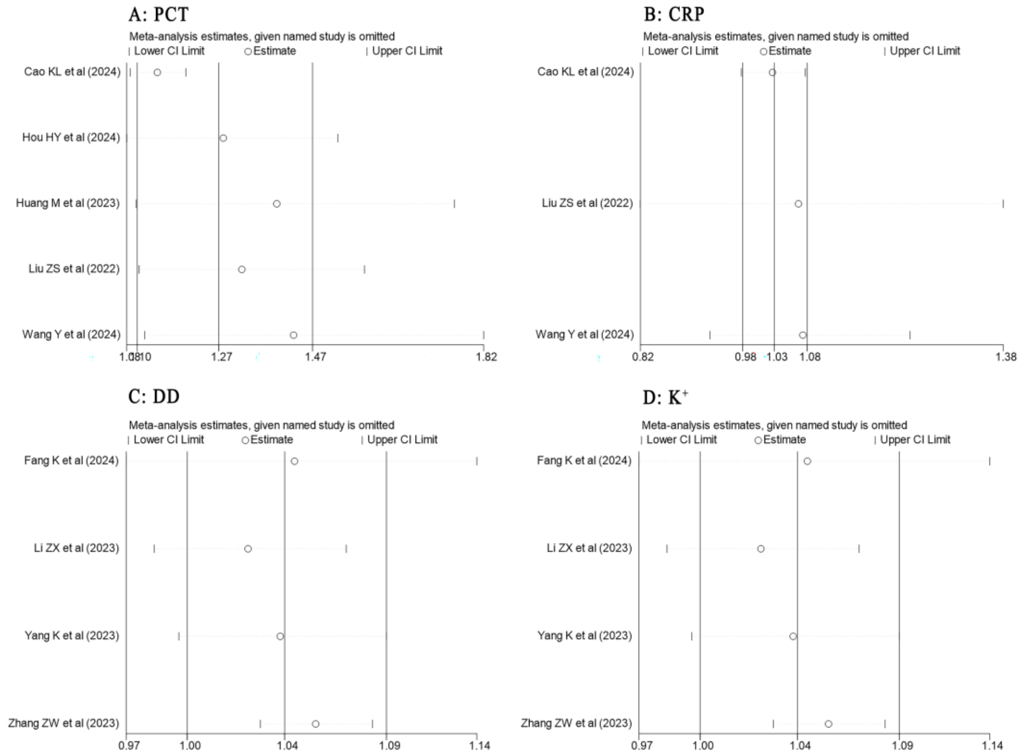

Figure S19. A sensitivity analysis for the literature related to other laboratory parameters.

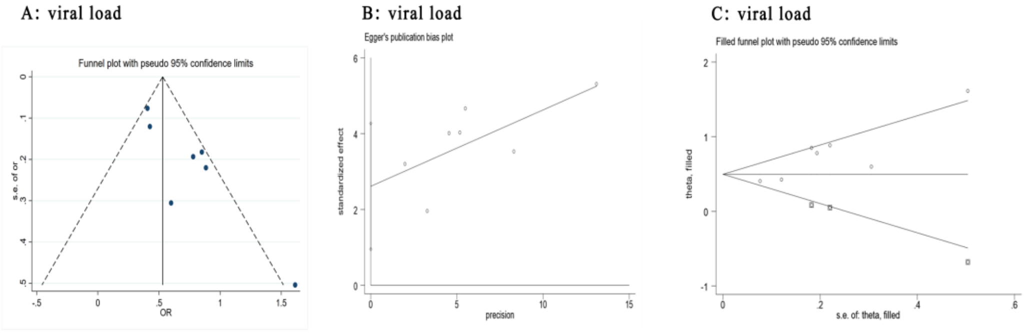

Figure S20. Publication bias assessment for viral load.

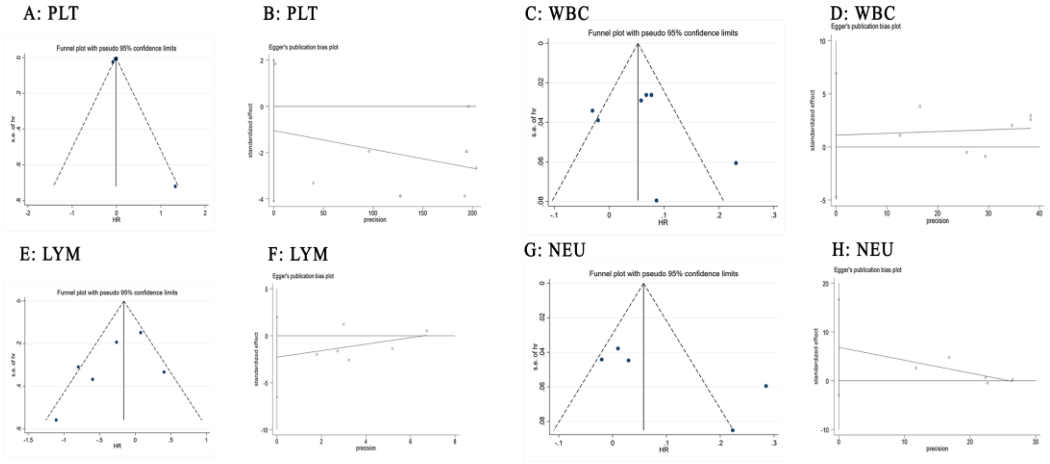

Figure S21. Publication bias assessment for blood routine indicators.

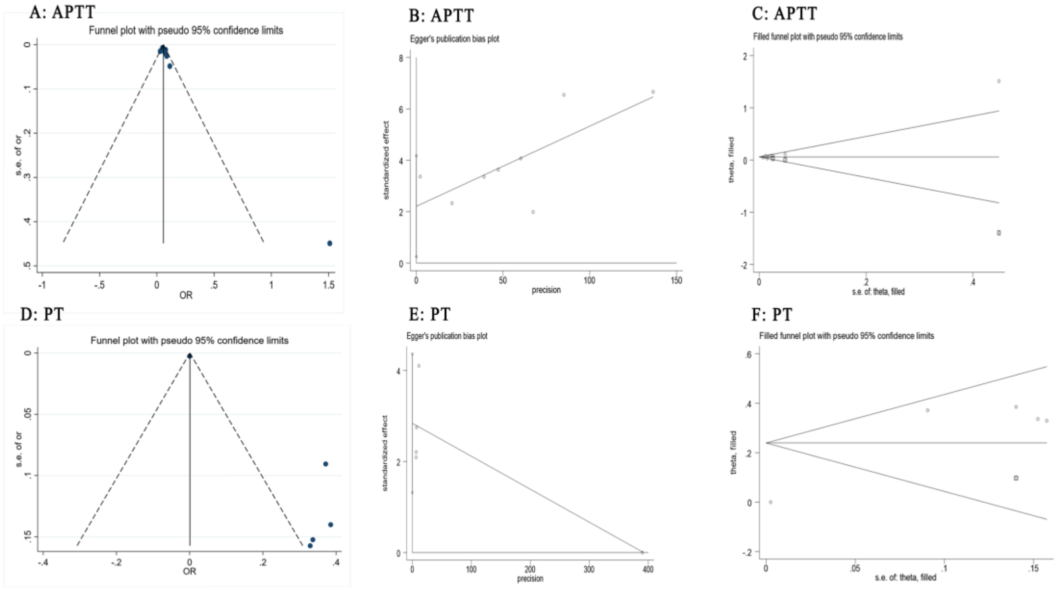

Figure S22. **Publication bias assessment for coagulation indicators.**

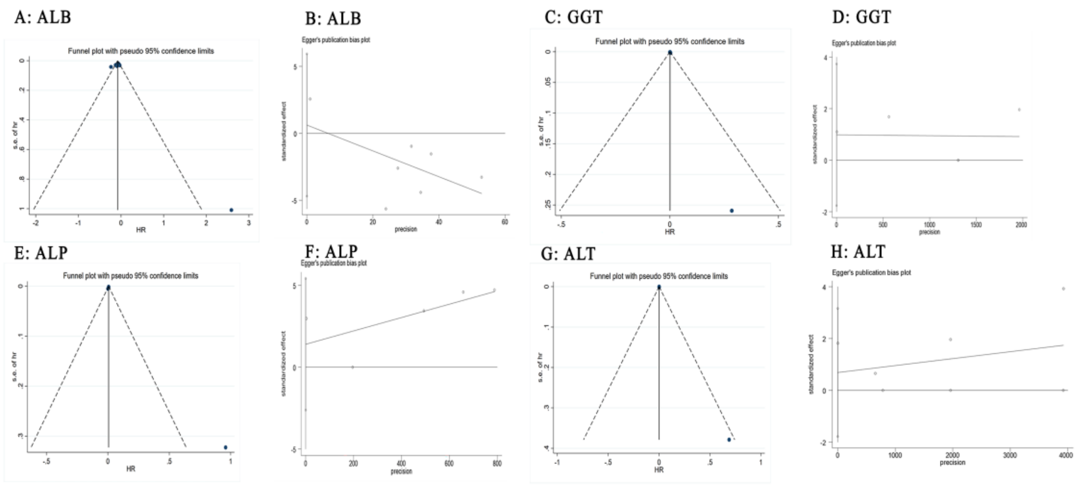

Figure S23. Publication bias assessment for liver function indicators.

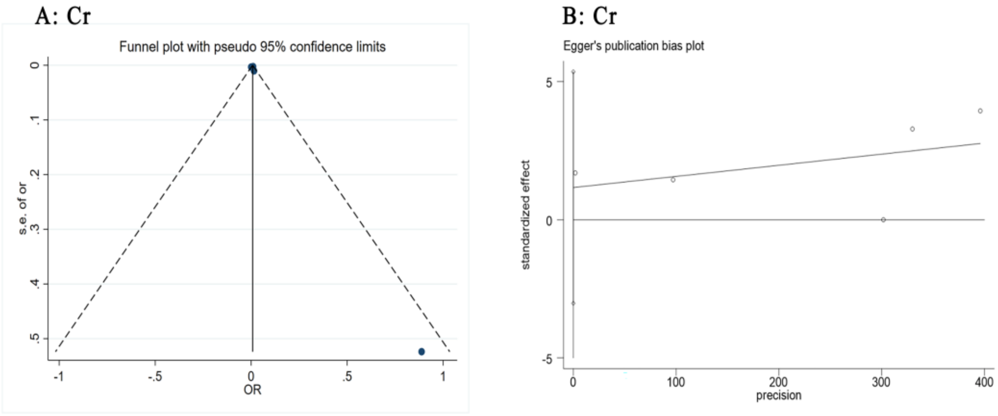

Figure S24. Publication bias assessment for renal function indicators.

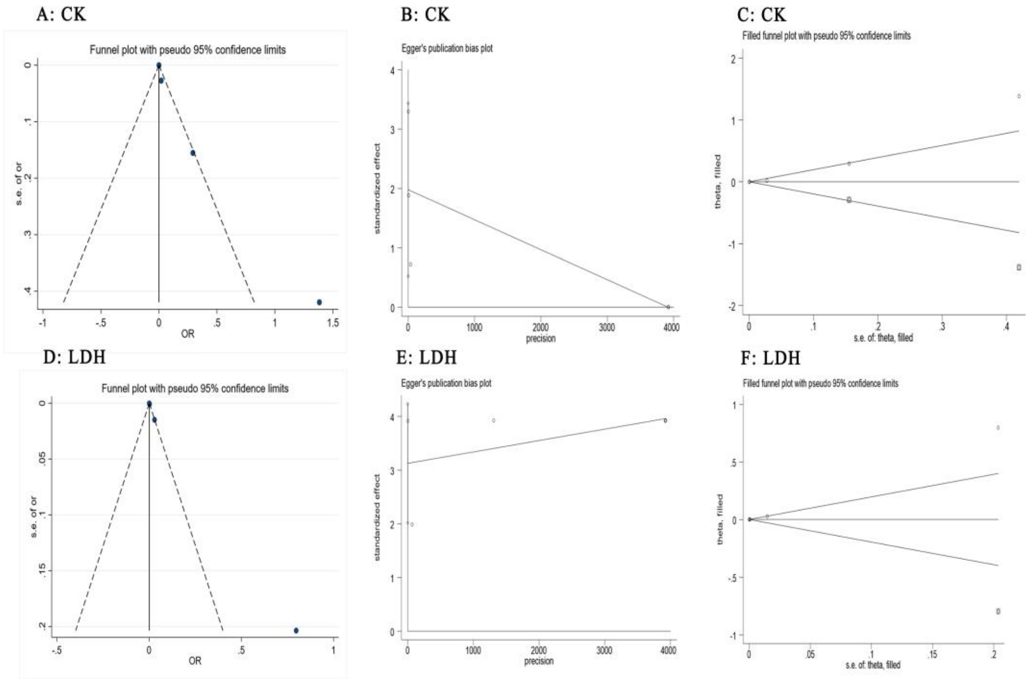

Figure S25. Publication bias assessment for myocardial function indicators.

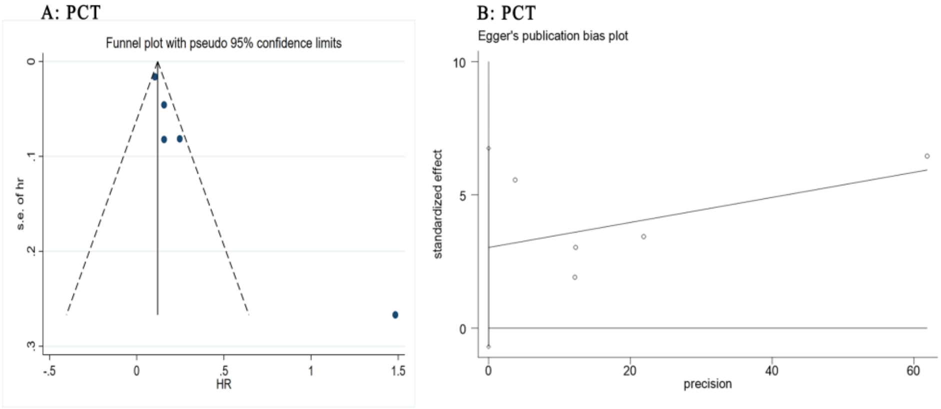

Figure S26. Publication bias assessment for other laboratory parameters.
