# Supplementary material for: Prediction of Mortality by Clinical Laboratory Parameters in Severe Fever with Thrombocytopenia Syndrome: A Meta-Analysis
Source: Trop Med Infect Dis. 2025 Jul 9;10(7):193. doi: 10.3390/tropicalmed10070193 (PMC12300845; doi:10.3390/tropicalmed10070193)
Supplement: Supplementary file 1 [file tropicalmed-10-00193-s001.zip › Table S2.pdf]

Table S2. Search strategy

Search History of Pubmed

Search number	Query	Results
1	(("Severe Fever with Thrombocytopenia Syndrome"[Mesh]) OR "Orthobunyavirus"[Mesh]) OR "Heartland virus" [Supplementary Concept]	2,486
2	"Apeu virus"[Title/Abstract] OR "bunya virus"[Title/Abstract] OR "Bunyavir*"[Title/Abstract] OR "Catu virus"[Title/Abstract] OR "bandavirus"[Title/Abstract] OR "Guama virus"[Title/Abstract] OR "Heartland bandavirus"[Title/Abstract] OR "Heartland banyangvirus"[Title/Abstract] OR "Heartland virus"[Title/Abstract] OR "HRTV virus"[Title/Abstract] OR "Orthobunyavirus*"[Title/Abstract] OR "Peribunyaviridae"[Title/Abstract] OR "severe fever with thrombocytopenia syndrome"[Title/Abstract] OR "SFTS*"[Title/Abstract] OR "Dabie Banda Virus"[Title/Abstract] OR "DBV"[Title/Abstract] OR "Phenuiviridae"[Title/Abstract]	5,349
3	#1 OR #2	6,414
4	((((((((((("Aspartate Aminotransferases"[Mesh]) OR "Alanine Transaminase"[Mesh]) OR "C-Reactive Protein"[Mesh]) OR "L-Lactate Dehydrogenase"[Mesh]) OR "Blood Platelets"[Mesh])	1,727,150

	OR "Leukocytes"[Mesh]) OR "Creatine Kinase"[Mesh]) OR "fibrin fragment D" [Supplementary Concept]) OR "Amylases"[Mesh]) OR "Ferritins"[Mesh]) OR "Lipase"[Mesh]) OR "Cytokines"[Mesh]) OR "Interleukin-6"[Mesh]	
5	"26 k protein"[Title/Abstract] OR "ADP Phosphocreatine Phosphotransferase"[Title/Abstract] OR "AIF 1"[Title/Abstract] OR "AIF1"[Title/Abstract] OR "alanin* aminotransferase"[Title/Abstract] OR "alanine amino transferase"[Title/Abstract] OR "alanine transaminase"[Title/Abstract] OR "alanine transpeptidase"[Title/Abstract] OR "allograft inflammatory factor 1"[Title/Abstract] OR "alpha maxilase"[Title/Abstract] OR "ALT"[Title/Abstract] OR "amylase*"[Title/Abstract] OR "amylesterase"[Title/Abstract] OR "amylolysis"[Title/Abstract] OR "amylolytic enzyme"[Title/Abstract] OR "amylolytic hydrolase"[Title/Abstract] OR "amylopsin"[Title/Abstract] OR "Aspart* Aminotransferase*"[Title/Abstract] OR "Aspart* Transaminase"[Title/Abstract] OR "aspartate amino transferase"[Title/Abstract] OR "Aspartate Apoaminotransferase"[Title/Abstract] OR "AST"[Title/Abstract] OR "B Cell Differentiation Factor"[Title/Abstract] OR "b cell stimulat* factor 2"[Title/Abstract] OR "b lymphocyte stimulating	1,550,503

factor 2"[Title/Abstract] OR "Basic Isoferritin"[Title/Abstract] OR "beta 2 interferon"[Title/Abstract] OR "beta2 interferon"[Title/Abstract] OR "BSF 2"[Title/Abstract] OR "bsf2"[Title/Abstract] OR "buclamase"[Title/Abstract] OR "c reaction protein"[Title/Abstract] OR "c reactive protein"[Title/Abstract] OR "CK"[Title/Abstract] OR "CPK"[Title/Abstract] OR "creactive protein"[Title/Abstract] OR "Creatine Kinase"[Title/Abstract] OR "Creatine Phosphokinase"[Title/Abstract] OR "creatine phosphotransferase"[Title/Abstract] OR "creatinekinase"[Title/Abstract] OR "creatinephosphokinase"[Title/Abstract] OR "creatinine kinase"[Title/Abstract] OR "creatinphosphokinase"[Title/Abstract] OR "crosslinked fibrin degradation product"[Title/Abstract] OR "CRP"[Title/Abstract] OR "Cytokine*"[Title/Abstract] OR "D D"[Title/Abstract] OR "D dimer"[Title/Abstract] OR "daintain"[Title/Abstract] OR "diastase"[Title/Abstract] OR "Exolipase"[Title/Abstract] OR "ferritin*"[Title/Abstract] OR "fibrin degradation product d dimer"[Title/Abstract] OR "fibrin fragment D*"[Title/Abstract] OR "fortizyme"[Title/Abstract] OR "glutam* oxalacetic transaminase"[Title/Abstract] OR "glutam* oxalic transaminase"[Title/Abstract] OR "glutam* pyruvate

transaminase"[Title/Abstract] OR "glutamate oxalacetate transaminase"[Title/Abstract] OR "glutamate oxalate transaminase"[Title/Abstract] OR "glutamate oxaloacetate aminotransferase"[Title/Abstract] OR "glutamate oxaloacetate transaminase"[Title/Abstract] OR "glutamate oxaloacetic acid transaminase"[Title/Abstract] OR "glutamate pyruvate aminotransferase"[Title/Abstract] OR "glutamate pyruvatetransaminase"[Title/Abstract] OR "glutamatoxalacetate transaminase"[Title/Abstract] OR "glutamic oxal acetatic transaminase"[Title/Abstract] OR "glutamic oxalacetic transferase"[Title/Abstract] OR "glutamic oxaloacetic aminotransferase"[Title/Abstract] OR "Glutam* Oxaloacetic Transaminase"[Title/Abstract] OR "glutamic pyruvic aminotransferase"[Title/Abstract] OR "Glutamic Pyruvic Transaminase"[Title/Abstract] OR "glutamopyruvic transaminase"[Title/Abstract] OR "glycogenase"[Title/Abstract] OR "GOT"[Title/Abstract] OR "GPT"[Title/Abstract] OR "hepatocyte stimulating factor"[Title/Abstract] OR "horse ferritin"[Title/Abstract] OR "hsCRP"[Title/Abstract] OR "human leukocyte"[Title/Abstract] OR "Hybridoma Growth Factor"[Title/Abstract] OR "IFN beta 2"[Title/Abstract] OR "IL 6"[Title/Abstract] OR "IL6"[Title/Abstract] OR

"immunoferritin"[Title/Abstract] OR "inflammatory cytokines"[Title/Abstract] OR "inflammatory factor"[Title/Abstract] OR "Interferon beta 2"[Title/Abstract] OR "interferon beta2"[Title/Abstract] OR "interleukin 6"[Title/Abstract] OR "interleukin b"[Title/Abstract] OR "interleukin hp1"[Title/Abstract] OR "Isoferritin"[Title/Abstract] OR "koji"[Title/Abstract] OR "l lactate nad oxidoreductase"[Title/Abstract] OR "l lactate nicotinamide adenine dinucleotide oxidoreductase"[Title/Abstract] OR "Lactate Dehydrogenase*"[Title/Abstract] OR "lactatedehydrogenase"[Title/Abstract] OR "lactic dehydrogenase"[Title/Abstract] OR "lacticodehydrogenase"[Title/Abstract] OR "lactodehydrogenase"[Title/Abstract] OR "LDH"[Title/Abstract] OR "leucocyte*"[Title/Abstract] OR "Leukocyte*"[Title/Abstract] OR "Lipase"[Title/Abstract] OR "lipid hydrolase"[Title/Abstract] OR "lipidase"[Title/Abstract] OR "liver cell stimulating factor"[Title/Abstract] OR "Macro Creatine Kinase"[Title/Abstract] OR "maxilase"[Title/Abstract] OR "MGI 2"[Title/Abstract] OR "mycoferritin"[Title/Abstract] OR "Myeloid Differentiation Inducing Protein"[Title/Abstract] OR "pancreatolipase"[Title/Abstract] OR "peripheral leu*ocyte"[Title/Abstract] OR "phosphocreatine

	kinase"[Title/Abstract] OR "phosphocreatinkinase"[Title/Abstract] OR "plasmacytoma growth factor"[Title/Abstract] OR "platelet*"[Title/Abstract] OR "PLT"[Title/Abstract] OR "protein 26k"[Title/Abstract] OR "proteoferrin"[Title/Abstract] OR "sanifer"[Title/Abstract] OR "SGOT"[Title/Abstract] OR "SGPT"[Title/Abstract] OR "steapsin"[Title/Abstract] OR "taka amylase"[Title/Abstract] OR "thrombocyte*"[Title/Abstract] OR "transaminase a"[Title/Abstract] OR "triacylglycerol acylhydrolase"[Title/Abstract] OR "triacylglycerol hydrolase"[Title/Abstract] OR "tributyrase"[Title/Abstract] OR "tributyrinase"[Title/Abstract] OR "triglycerid*"[Title/Abstract] OR "Triolean Hydrolase"[Title/Abstract] OR "uman leucocyte"[Title/Abstract] OR "unifer"[Title/Abstract] OR "WBC"[Title/Abstract] OR "White Blood Corpuscle*"[Title/Abstract] OR "white cell*"[Title/Abstract] OR "zymoplex"[Title/Abstract]	
6	#4 OR #5	2,561,565
7	#3 AND #6	564

Search History of Cochrane

ID	Search	Hits

#1	MeSH descriptor: [Severe Fever with Thrombocytopenia Syndrome] explode all trees	4
#2	MeSH descriptor: [Orthobunyavirus] explode all trees	2
#3	MeSH descriptor: [Heartland virus] explode all trees	0
#4	('Apeu virus' OR 'bunya virus' OR 'Bunyavir*' OR 'Catu virus' OR 'bandavirus' OR 'Guama virus' OR 'Heartland bandavirus' OR 'Heartland banyangvirus' OR 'Heartland virus' OR 'HRTV virus' OR 'Orthobunyavirus*' OR 'Peribunyaviridae' OR 'severe fever with thrombocytopenia syndrome' OR 'SFTS*' OR 'Dabie Banda Virus' OR 'DBV' OR 'Phenuiviridae'):ti,kw,ab	113
#5	#1 OR #2 OR #3 OR #4	115
#6	MeSH descriptor: [Aspartate Aminotransferases] explode all trees	1278
#7	MeSH descriptor: [Alanine Transaminase] explode all trees	2035
#8	MeSH descriptor: [C-Reactive Protein] explode all trees	6525
#9	MeSH descriptor: [L-Lactate Dehydrogenase] explode all trees	503
#10	MeSH descriptor: [Blood Platelets] explode all trees	2543
#11	MeSH descriptor: [Leukocytes] explode all trees	12878
#12	MeSH descriptor: [Creatine Kinase] explode all trees	1909
#13	MeSH descriptor: [Ferritins] explode all trees	1385
#14	MeSH descriptor: [fibrin fragment D] explode all trees	0
#15	MeSH descriptor: [Amylases] explode all trees	634
#16	MeSH descriptor: [Lipase] explode all trees	629

#17	MeSH descriptor: [Cytokines] explode all trees	28020
#18	MeSH descriptor: [Interleukin-6] explode all trees	4509
#19	('26 k protein' OR 'ADP Phosphocreatine Phosphotransferase' OR 'AIF 1' OR 'AIF1' OR 'alanin* aminotransferase' OR 'alanine amino transferase' OR 'alanine transaminase' OR 'alanine transpeptidase' OR 'allograft inflammatory factor 1' OR 'alpha maxilase' OR 'ALT' OR 'amylase*' OR 'amylesterase' OR 'amylolysis' OR 'amylolytic enzyme' OR 'amylolytic hydrolase' OR 'amylopsin' OR 'Aspart* Aminotransferase*' OR 'Aspart* Transaminase' OR 'aspartate amino transferase' OR 'Aspartate Apoaminotransferase' OR 'AST' OR 'B Cell Differentiation Factor' OR 'b cell stimulat* factor 2' OR 'b lymphocyte stimulating factor 2' OR 'Basic Isoferritin' OR 'beta 2 interferon' OR 'beta2 interferon' OR 'BSF 2' OR 'bsf2' OR 'buclamase' OR 'c reaction protein' OR 'c reactive protein' OR 'CK' OR 'CPK' OR 'creactive protein' OR 'Creatine Kinase' OR 'Creatine Phosphokinase' OR 'creatine phosphotransferase' OR 'creatinekinase' OR 'creatinephosphokinase' OR 'creatinine kinase' OR 'creatinphosphokinase' OR 'crosslinked fibrin degradation product' OR 'CRP' OR 'Cytokine*' OR 'D D' OR 'D dimer' OR 'daintain' OR 'diastase' OR 'Exolipase' OR 'ferritin*' OR 'fibrin degradation product d dimer' OR 'fibrin fragment D*' OR 'fortizyme' OR 'glutam* oxalacetic transaminase' OR 'glutam* oxalic transaminase'	315356

OR 'glutam* pyruvate transaminase' OR 'glutamate oxalacetate transaminase' OR 'glutamate oxalate transaminase' OR 'glutamate oxaloacetate aminotransferase' OR 'glutamate oxaloacetate transaminase' OR 'glutamate oxaloacetic acid transaminase' OR 'glutamate pyruvate aminotransferase' OR 'glutamate pyruvatetransaminase' OR 'glutamatoxalacetate transaminase' OR 'glutamic oxal acetatic transaminase' OR 'glutamic oxalacetic transferase' OR 'glutamic oxaloacetic aminotransferase' OR 'Glutam* Oxaloacetic Transaminase' OR 'glutamic pyruvic aminotransferase' OR 'Glutamic Pyruvic Transaminase' OR 'glutamopyruvic transaminase' OR 'glycogenase' OR 'GOT' OR 'GPT' OR 'hepatocyte stimulating factor' OR 'horse ferritin' OR 'hsCRP' OR 'human leukocyte' OR 'Hybridoma Growth Factor' OR 'IFN beta 2' OR 'IL 6' OR 'IL6' OR 'immunoferritin' OR 'inflammatory cytokines' OR 'inflammatory factor' OR 'Interferon beta 2' OR 'interferon beta2' OR 'interleukin 6' OR 'interleukin b' OR 'interleukin hp1' OR 'Isoferritin' OR 'koji' OR 'l lactate nad oxidoreductase' OR 'l lactate nicotinamide adenine dinucleotide oxidoreductase' OR 'Lactate Dehydrogenase*' OR 'lactatedehydrogenase' OR 'lactic dehydrogenase' OR 'lacticodehydrogenase' OR 'lactodehydrogenase' OR 'LDH' OR 'leucocyte*' OR 'Leukocyte*' OR 'Lipase' OR 'lipid hydrolase' OR

	'lipidase' OR 'liver cell stimulating factor' OR 'Macro Creatine Kinase' OR 'maxilase' OR 'MGI 2' OR 'mycoferritin' OR 'Myeloid Differentiation Inducing Protein' OR 'pancreatolipase' OR 'peripheral leu*ocyte' OR 'phosphocreatine kinase' OR 'phosphocreatinkinase' OR 'plasmacytoma growth factor' OR 'platelet*' OR 'PLT' OR 'protein 26k' OR 'proteoferrin' OR 'sanifer' OR 'SGOT' OR 'SGPT' OR 'steapsin' OR 'taka amylase' OR 'thrombocyte*' OR 'transaminase a' OR 'triacylglycerol acylhydrolase' OR 'triacylglycerol hydrolase' OR 'tributyrase' OR 'tributyrinase' OR 'triglycerid*' OR 'Triolean Hydrolase' OR 'uman leucocyte' OR 'unifer' OR 'WBC' OR 'White Blood Corpuscle*' OR 'white cell*' OR 'zymoplex'):ti,kw,ab	
#20	#6 OR #7 OR #8 OR #9 OR #10 OR #11 OR #12 OR #13 OR #14 OR #15 OR #16 OR #17 OR #18 OR #19	328818
#21	#5 AND #20	65

Search History of Embase

No.	Query	Results
1	'severe fever with thrombocytopenia syndrome'/exp	1007
2	'bunyaviridae'/exp	13498
3	'bandavirus'/exp	428
4	'phenuiviridae'/exp	534

5	'apeu virus':ti,kw,ab OR 'bunya virus':ti,kw,ab OR 'bunyavir*':ti,kw,ab OR 'catu virus':ti,kw,ab OR 'bandavirus':ti,kw,ab OR 'guama virus':ti,kw,ab OR 'heartland bandavirus':ti,kw,ab OR 'heartland banyangvirus':ti,kw,ab OR 'heartland virus':ti,kw,ab OR 'hrtv virus':ti,kw,ab OR 'orthobunyavirus*':ti,kw,ab OR 'peribunyaviridae':ti,kw,ab OR 'severe fever with thrombocytopenia syndrome':ti,kw,ab OR 'sfts*':ti,kw,ab OR 'dabie banda virus':ti,kw,ab OR 'dbv':ti,kw,ab OR 'phenuiviridae':ti,kw,ab	6047
6	#1 OR #2 OR #3 OR #4 OR #5	16403
7	'aspartate aminotransferase'/exp	168445
8	'alanine aminotransferase'/exp	189290
9	'c reactive protein'/exp	292060
10	'lactate dehydrogenase'/exp	148901
11	'thrombocyte'/exp	141802
12	'leukocyte'/exp	1606846
13	'creatine kinase'/exp	72297
14	'ferritin'/exp	72966
15	'd dimer'/exp	46635
16	'amylase'/exp	54192
17	'triacylglycerol lipase'/exp	50719
18	'allograft inflammatory factor 1'/exp	3220
19	'interleukin 6'/exp	374487

'26 k protein':ti,kw,ab OR 'adp phosphocreatine phosphotransferase':ti,kw,ab OR 'aif 1':ti,kw,ab OR 'aif1':ti,kw,ab OR 'alanin* aminotransferase':ti,kw,ab OR 'alanine amino transferase':ti,kw,ab OR 'alanine transaminase':ti,kw,ab OR 'alanine transpeptidase':ti,kw,ab OR 'allograft inflammatory factor 1':ti,kw,ab OR 'alpha maxilase':ti,kw,ab OR 'alt':ti,kw,ab OR 'amylase*':ti,kw,ab OR 'amylesterase':ti,kw,ab OR 'amylolysis':ti,kw,ab OR 'amylolytic enzyme':ti,kw,ab OR 'amylolytic hydrolase':ti,kw,ab OR 'amylopsin':ti,kw,ab OR 'aspart* aminotransferase*':ti,kw,ab OR 'aspart* transaminase':ti,kw,ab OR 'aspartate amino transferase':ti,kw,ab OR 'aspartate apoaminotransferase':ti,kw,ab OR

'ast':ti,kw,ab OR 'b cell differentiation factor':ti,kw,ab OR 'b cell stimulat* factor 2':ti,kw,ab OR 'b lymphocyte stimulating factor 2':ti,kw,ab OR 'basic isoferritin':ti,kw,ab OR 'beta 2 interferon':ti,kw,ab OR 'beta2 interferon':ti,kw,ab OR 'bsf 2':ti,kw,ab OR 'bsf2':ti,kw,ab OR 'buclamase':ti,kw,ab OR 'c reaction protein':ti,kw,ab OR 'c reactive protein':ti,kw,ab OR 'ck':ti,kw,ab OR 'cpk':ti,kw,ab OR 'creactive protein':ti,kw,ab OR 'creatine kinase':ti,kw,ab OR 'creatine phosphokinase':ti,kw,ab OR 'creatine phosphotransferase':ti,kw,ab OR 'creatinekinase':ti,kw,ab OR 'creatinephosphokinase':ti,kw,ab OR 'creatinine kinase':ti,kw,ab OR 'creatinphosphokinase':ti,kw,ab OR 'crosslinked fibrin degradation product':ti,kw,ab OR 'crp':ti,kw,ab OR

'cytokine*':ti,kw,ab OR 'd d':ti,kw,ab OR 'd dimer':ti,kw,ab OR 'daintain':ti,kw,ab OR 'diastase':ti,kw,ab OR 'exolipase':ti,kw,ab OR 'ferritin*':ti,kw,ab OR 'fibrin degradation product d dimer':ti,kw,ab OR 'fibrin fragment d*':ti,kw,ab OR 'fortizyme':ti,kw,ab OR 'glutam* oxalacetic transaminase':ti,kw,ab OR 'glutam* oxalic transaminase':ti,kw,ab OR 'glutam* pyruvate transaminase':ti,kw,ab OR 'glutamate oxalacetate transaminase':ti,kw,ab OR 'glutamate oxalate transaminase':ti,kw,ab OR 'glutamate oxaloacetate aminotransferase':ti,kw,ab OR 'glutamate oxaloacetate transaminase':ti,kw,ab OR 'glutamate oxaloacetic acid transaminase':ti,kw,ab OR 'glutamate pyruvate aminotransferase':ti,kw,ab OR 'glutamate pyruvatetransaminase':ti,kw,ab OR 'glutamatoxalacetate transaminase':ti,kw,ab OR 'glutamic oxal acetatic transaminase':ti,kw,ab OR 'glutamic oxalacetic transferase':ti,kw,ab OR 'glutamic oxaloacetic aminotransferase':ti,kw,ab OR 'glutam* oxaloacetic transaminase':ti,kw,ab OR 'glutamic pyruvic aminotransferase':ti,kw,ab OR 'glutamic pyruvic transaminase':ti,kw,ab OR 'glutamopyruvic transaminase':ti,kw,ab OR 'glycogenase':ti,kw,ab OR 'got':ti,kw,ab OR 'gpt':ti,kw,ab OR 'hepatocyte stimulating factor':ti,kw,ab OR 'horse ferritin':ti,kw,ab OR 'hscrp':ti,kw,ab OR 'human leukocyte':ti,kw,ab OR 'hybridoma growth factor':ti,kw,ab OR

'ifn beta 2':ti,kw,ab OR 'il 6':ti,kw,ab OR 'il6':ti,kw,ab OR 'immunoferritin':ti,kw,ab OR 'inflammatory cytokines':ti,kw,ab OR 'inflammatory factor':ti,kw,ab OR 'interferon beta 2':ti,kw,ab OR 'interferon beta2':ti,kw,ab OR 'interleukin 6':ti,kw,ab OR 'interleukin b':ti,kw,ab OR 'interleukin hp1':ti,kw,ab OR 'isoferritin':ti,kw,ab OR 'koji':ti,kw,ab OR 'l lactate nad oxidoreductase':ti,kw,ab OR 'l lactate nicotinamide adenine dinucleotide oxidoreductase':ti,kw,ab OR 'lactate dehydrogenase*':ti,kw,ab OR 'lactatedehydrogenase':ti,kw,ab OR 'lactic dehydrogenase':ti,kw,ab OR 'lacticodehydrogenase':ti,kw,ab OR 'lactodehydrogenase':ti,kw,ab OR 'ldh':ti,kw,ab OR 'leucocyte*':ti,kw,ab OR 'leukocyte*':ti,kw,ab OR 'lipase':ti,kw,ab OR 'lipid hydrolase':ti,kw,ab OR 'lipidase':ti,kw,ab OR 'liver cell stimulating factor':ti,kw,ab OR 'macro creatine kinase':ti,kw,ab OR 'maxilase':ti,kw,ab OR 'mgi 2':ti,kw,ab OR 'mycoferritin':ti,kw,ab OR 'myeloid differentiation inducing protein':ti,kw,ab OR 'pancreatolipase':ti,kw,ab OR 'peripheral leu*ocyte':ti,kw,ab OR 'phosphocreatine kinase':ti,kw,ab OR 'phosphocreatinkinase':ti,kw,ab OR 'plasmacytoma growth factor':ti,kw,ab OR 'platelet*':ti,kw,ab OR 'plt':ti,kw,ab OR 'protein 26k':ti,kw,ab OR 'proteoferrin':ti,kw,ab OR 'sanifer':ti,kw,ab OR 'sgot':ti,kw,ab OR 'sgpt':ti,kw,ab OR 'steapsin':ti,kw,ab OR 'taka amylase':ti,kw,ab OR 'thrombocyte*':ti,kw,ab OR 'transaminase a':ti,kw,ab OR

	'triacylglycerol acylhydrolase':ti,kw,ab OR 'triacylglycerol hydrolase':ti,kw,ab OR 'tributyrase':ti,kw,ab OR 'tributyrinase':ti,kw,ab OR 'triglycerid*':ti,kw,ab OR 'triolean hydrolase':ti,kw,ab OR 'uman leucocyte':ti,kw,ab OR 'unifer':ti,kw,ab OR 'wbc':ti,kw,ab OR 'white blood corpuscle*':ti,kw,ab OR 'white cell*':ti,kw,ab OR 'zymoplex':ti,kw,ab	
21	#7 OR #8 OR #9 OR #10 OR #11 OR #12 OR #13 OR #14 OR #15 OR #16 OR #17 OR #18 OR #19 OR #20	3727529
22	#6 AND #21	1857

Search History of Web of Science

#	Search Query	Results
1	TS=((Apeu virus) OR (bunya virus) OR (Bunyavir*) OR (Catu virus) OR (bandavirus) OR (Guama virus) OR (Heartland bandavirus) OR (Heartland banyangvirus) OR (Heartland virus) OR (HRTV virus) OR (Orthobunyavirus*) OR (Peribunyaviridae) OR (severe fever with thrombocytopenia syndrome) OR (SFTS*) OR (Dabie Banda Virus) OR (DBV) OR (Phenuiviridae))	6671
2	TS=((26 k protein) OR (ADP Phosphocreatine Phosphotransferase) OR (AIF 1) OR (AIF1) OR (alanin* aminotransferase) OR (alanine amino transferase) OR (alanine transaminase) OR (alanine transpeptidase) OR (allograft inflammatory factor 1) OR (alpha maxilase) OR (ALT) OR	4520355

(amylase*) OR (amylesterase) OR (amylolysis) OR (amylolytic enzyme) OR (amylolytic hydrolase) OR (amylopsin) OR (Aspart* Aminotransferase*) OR (Aspart* Transaminase) OR (aspartate amino transferase) OR (Aspartate Apoaminotransferase) OR (AST) OR (B Cell Differentiation Factor) OR (b cell stimulat* factor 2) OR (b lymphocyte stimulating factor 2) OR (Basic Isoferritin) OR (beta 2 interferon) OR (beta2 interferon) OR (BSF 2) OR (bsf2) OR (buclamase) OR (c reaction protein) OR (c reactive protein) OR (CK) OR (CPK) OR (creactive protein) OR (Creatine Kinase) OR (Creatine Phosphokinase) OR (creatine phosphotransferase) OR (creatinekinase) OR (creatinephosphokinase) OR (creatinine kinase) OR (creatinphosphokinase) OR (crosslinked fibrin degradation product) OR (CRP) OR (Cytokine*) OR (D D) OR (D dimer) OR (daintain) OR (diastase) OR (Exolipase) OR (ferritin*) OR (fibrin degradation product d dimer) OR (fibrin fragment D) OR (fibrin fragment D1 dimer) OR (fibrin fragment DD) OR (fortizyme) OR (glutam* oxalacetic transaminase) OR (glutam* oxalic transaminase) OR (glutam* pyruvate transaminase) OR (glutamate oxalacetate transaminase) OR (glutamate oxalate transaminase) OR (glutamate oxaloacetate aminotransferase) OR (glutamate oxaloacetate transaminase) OR (glutamate oxaloacetic acid transaminase) OR (glutamate pyruvate aminotransferase) OR (glutamate pyruvatetransaminase) OR (glutamatoxalacetate transaminase) OR (glutamic oxal acetatic transaminase) OR (glutamic

oxalacetic transferase) OR (glutamic oxaloacetic aminotransferase) OR (Glutam* Oxaloacetic Transaminase) OR (glutamic pyruvic aminotransferase) OR (Glutamic Pyruvic Transaminase) OR (glutamopyruvic transaminase) OR (glycogenase) OR (GOT) OR (GPT) OR (hepatocyte stimulating factor) OR (horse ferritin) OR (hsCRP) OR (human leukocyte) OR (Hybridoma Growth Factor) OR (IFN beta 2) OR (IL 6) OR (IL6) OR (immunoferritin) OR (inflammatory cytokines) OR (inflammatory factor) OR (Interferon beta 2) OR (interferon beta2) OR (interleukin 6) OR (interleukin b) OR (interleukin hp1) OR (Isoferritin) OR (koji) OR (l lactate nad oxidoreductase) OR (l lactate nicotinamide adenine dinucleotide oxidoreductase) OR (Lactate Dehydrogenase*) OR (lactatedehydrogenase) OR (lactic dehydrogenase) OR (lacticodehydrogenase) OR (lactodehydrogenase) OR (LDH) OR (leucocyte*) OR (Leukocyte*) OR (Lipase) OR (lipid hydrolase) OR (lipidase) OR (liver cell stimulating factor) OR (Macro Creatine Kinase) OR (maxilase) OR (MGI 2) OR (mycoferritin) OR (Myeloid Differentiation Inducing Protein) OR (pancreatolipase) OR (peripheral leu*ocyte) OR (phosphocreatine kinase) OR (phosphocreatinkinase) OR (plasmacytoma growth factor) OR (platelet*) OR (PLT) OR (protein 26k) OR (proteoferrin) OR (sanifer) OR (SGOT) OR (SGPT) OR (steapsin) OR (taka amylase) OR (thrombocyte*) OR (transaminase a) OR (triacylglycerol acylhydrolase) OR (triacylglycerol hydrolase) OR (tributyrase) OR (tributyrinase) OR (triglycerid*) OR

	(Triolean Hydrolase) OR (uman leucocyte) OR (unifer) OR (WBC) OR (White Blood Corpuscle*) OR (white cell*) OR (zymoplex))	
3	#1 AND #2	1078
