# Supplementary material for: Prediction of Mortality by Clinical Laboratory Parameters in Severe Fever with Thrombocytopenia Syndrome: A Meta-Analysis
Source: Trop Med Infect Dis. 2025 Jul 9;10(7):193. doi: 10.3390/tropicalmed10070193 (PMC12300845; doi:10.3390/tropicalmed10070193)
Supplement: Supplementary file 1 [file tropicalmed-10-00193-s001.zip › Table S3.pdf]

Table S3. Newcastle-Ottawa Quality Assessment Scale of the 30 cohort studies.

Study	Selection of Participants				Comparability	Outcome				
	Representativeness of the Exposed Cohort	Selection of the Non-Exposed Cohort	Ascertainment of Exposure	Demonstration That Outcome of Interest Was Not Present at Start of Study	Comparability of Cohorts on the Basis of the Design or Analysis	Assessment of Outcome	Was Follow-Up Long Enough for Outcomes to Occur	Adequacy of Follow Up of Cohorts	Total score	Grade
Cao KL et al.2024	1	1	1	1	2	1	1	0	8	High
Guo CX et al.2024	1	1	1	1	2	1	0	0	7	High
Hou HY et al.,2024	1	1	1	1	2	1	1	0	8	High
Huang M et al.,2023	1	1	1	1	2	1	1	0	8	High
Kim JY et al.,2023	1	1	1	1	2	1	0	0	7	High

Li JN et al.,2024	1	1	1	1	2	1	1	0	8	High
Li ZX et al.,2023	1	1	1	1	2	1	0	0	7	High
Liang BY et al.,2024	1	1	1	1	2	1	0	0	7	High
Liu Y et al.,2022	1	1	1	1	1	1	1	0	7	High
Liu ZS et al.,2022	1	1	1	1	2	1	0	0	7	High
Peng WJ et al.,2024	1	1	1	1	2	1	1	0	8	High
Qian F et al.,2023	1	1	1	1	1	1	1	0	7	High
Wang DC et al.,2022	1	1	1	1	2	1	0	0	7	High
Wang F et al.,2020	1	1	1	1	2	1	0	0	7	High

Wang L et al.,2019	1	1	1	1	2	1	1	0	8	High
Wang L et al.,2024	1	1	1	1	2	1	0	0	7	High
Wang Y et al.,2024	1	1	1	1	2	1	0	0	7	High
Wei YY et al.,2022	1	1	1	1	1	1	1	0	7	High
Xia GM et al.,2023	1	1	1	1	2	1	0	0	7	High
Xiao WY et al.,2024	1	1	1	1	2	1	0	0	7	High
Xiong S et al.,2016	1	1	1	1	2	1	0	0	7	High
Yang B et al.,2017	1	1	1	1	2	1	0	0	7	High
Yang K	1	1	1	1	2	1	0	0	7	High

et al.,2023										
Zhang Q et al.,2024	1	1	1	1	2	1	0	0	7	High
Zhang SQ et al.,2024	1	1	1	1	2	1	0	0	7	High
Zhang SQ et al.,2024	1	1	1	1	2	1	1	0	8	High
Zhang Y et al.,2024	1	1	1	1	2	1	0	0	7	High
Zhang ZW et al.,2023	1	1	1	1	2	1	0	0	7	High
Zhang ZW et al.,2024	1	1	1	1	2	1	0	0	7	High
Zhong F et al.,2024	1	1	1	1	1	1	1	0	7	High
