# Supplementary material for: Prediction of Mortality by Clinical Laboratory Parameters in Severe Fever with Thrombocytopenia Syndrome: A Meta-Analysis
Source: Trop Med Infect Dis. 2025 Jul 9;10(7):193. doi: 10.3390/tropicalmed10070193 (PMC12300845; doi:10.3390/tropicalmed10070193)
Supplement: Supplementary file 1 [file tropicalmed-10-00193-s001.zip › Table S4.pdf]

Table S4. Newcastle-Ottawa Quality Assessment Scale of the 3 case control studies.

study	Selection of Participants				Comparability	Measurement of exposure				
	Is the Case Definition Adequate?	Representativeness of the Cases	Selection of Controls	Definition of Controls	Comparability of Cases and Controls on the Basis of the Design or Analysis	Ascertainment of Exposure	The exposure factors of the cases and controls were determined by the same method	Non-Response Rate	Total score	Grade
Fang K et al.2024	1	1	1	1	1	1	1	0	7	High
Liu ZS et al.,2022	1	1	1	1	1	1	1	0	7	High
Yang K et al.,2024	1	1	1	1	1	1	1	0	7	High
